# Reaching Out for Food: How Food Incentives Modulate Peripersonal Space Perception

**DOI:** 10.5334/joc.148

**Published:** 2021-03-10

**Authors:** Matias Bertonatti, Mathias Weymar, Werner Sommer, Martin H. Fischer

**Affiliations:** 1 Institut für Psychologie, Humboldt-Universität zu Berlin, Germany; 2 Department of Biological Psychology and Affective Science, Faculty of Human Sciences, University of Potsdam, Germany; 3 Faculty of Health Sciences Brandenburg, University of Potsdam, Germany; 4 Department of Cognitive Sciences, University of Potsdam, Germany

**Keywords:** embodied cognition, peripersonal space, event-related potentials (ERPs), food pictures, attention, motivation, reward

## Abstract

Two experiments were conducted to determine, first, whether food items influence participants’ estimations of the size of their subjective peripersonal space. It was of particular interest whether this representation is influenced by satiated/hungry states and is differentially affected by valence and calorie content of depicted stimuli. Second, event-related brain potentials (ERPs) were used, in order to obtain information about the time course of the observed effects and how they depend on the spatial location of the food pictures. For that purpose, participants had to decide whether food items shown at various distances along a horizontal plane in front of them, were reachable or not. In Experiment 1, when participants were hungry, they perceived an increase of their peripersonal space modulated by high-calorie items which were experienced as being more reachable than low-calorie items. In Experiment 2, the reachability findings were replicated and early and late components of ERPs showed an attentional enhancement in far space for food items when participants were hungry. These findings suggest that participants’ subjective peripersonal space increased while being hungry, especially for high-calorie contents. Attention also seems to be oriented more strongly to far space items due to their expected incentive-related salience, expanding the subjective representation of peripersonal space.

## Introduction

One goal of cognitive scientists is to understand the workings of the human mind. A recent paradigmatic change in this effort has been towards “embodied cognition” (Shapiro, 2019). This theoretical perspective places the human body into focus when studying the mind. As a result of evolution, our bodily existence imposes various constraints on how the mind works when we interact with our environment. We have evolved to select or prefer objects or persons that ensure our survival. For example, we prefer objects that can be used easily or as tools to enhance our movement efficiency. Similarly (and to the detriment of our health in a world of affluence), we often prefer high-calorie foods because they provide high levels of energy to support our survival efforts (e.g., Killgore et al., 2003).

The cognitive mechanisms of object selection and interaction have been studied in considerable detail from an embodied cognition perspective. The space around our body that can be reached (by hand) is generally defined as our “peripersonal space”, in contrast to “extrapersonal space” as the area into which we cannot reach (Coello et al., 2003, 2008, 2012; Fischer, 2000, 2005; Rizzolatti et al., 1981, 1997). Contrary to intuition, the boundary between peri- and extrapersonal space is not fixed or clearly perceptible but depends on several factors. Coello et al. (2012) found, when the threatening parts of dangerous objects (e.g., the blade of a cutter or the tip of a syringe) were oriented towards participants, their judgment of object reachability was reduced by 1.6 cm as compared to not-dangerous objects, indicating a shrinking of subjective peripersonal space. Other studies showed an extension of perceived peripersonal space after participants had briefly used tools for reaching objects (Berti & Frassinetti, 2000; Bourgeois et al., 2014; Pegna et al., 2001). Such changes were also seen in increased receptive fields of neurons in intraparietal cortex (Iriki et al., 2001). Also, reward expectations in relation to motor actions influenced peripersonal space representation (Gigliotti et al., 2019) in humans, indicating that we simulate the actions we plan to perform on objects.

Studies of the time course of our assessment of whether an object is within or outside of reach have revealed a biphasic pattern. It was shown that graspable objects drew more attention when they appeared in the lower visual field near the dominant hands of their right-handed participants, presumably because the objects were processed for action planning (Handy et al., 2003). This was indicated by an increased early positivity of the event-related brain potentials (ERP) around 100 ms after stimulus onset (the P1 component), which is an established marker for attention allocation (Mangun & Hillyard, 1991; Mangun, 1995). Also, reachable objects in peripersonal space were found to elicit larger amplitudes in an early negative-going ERP component (N1, 150–200 ms) but also in the late positive component (LPC, around 350 ms), than objects presented in extrapersonal space, indicating enhanced attention and more elaborate processing for reachable objects (Valdés-Conroy et al., 2014).

In contrast to the influence of our sensory-motor experiences on object perception and its assessment for action, the embodied nature of food perception has been somewhat neglected, although much is already known about how the mind and brain respond to food more generally. We review some of these facts before introducing a method to evaluate the impact of food objects on the subjective perception of peripersonal space.

Judgments and choices about food depend on many factors, among others, on environmental and psychosocial variables, such as exposure to food advertisements, and nutrition-related knowledge and habits (e.g., Stein et al., 2013). Food choice starts with expectations about the attributes of the food and the consequences of its consumption, indicating that we simulate the complete interaction with the food (Ohla et al., 2012; Kringelbach & Berridge, 2017). Thus, the process through which some stimuli are preferred instead of others is identified as selective attention (Dummel & Hübner, 2017). Attentional effects are regulated by the observer’s motivational state (cf. also Derryberry & Tucker, 1994). For example, Killgore et al. (2003) found that, compared to object pictures, high-calorie content food pictures specifically activated brain regions implicated in attention and motivation. Based on previous learning we attribute to each food item a reward value that influences the amount of attention allocated to it (Rogers & Hardman, 2015; Dummel & Hübner, 2017), resulting in attentive perception as well as motor impulses to obtain the food (Schur et al., 2009; Papies, Stroebe, & Aarts, 2008).

ERP studies have revealed the time-course of food evaluation in detail, showing biphasic processing in early and late ERP components. For instance, Schacht et al. (2016) recently reported that high valence food items induced larger P1 amplitudes than neutral ones and found effects of high valence food items on the LPC around 300–500 ms after stimulus onset. The LPC generally reflects the activation of the motivational system (appetitive or defensive), which also engages selective attention and sustained perceptual processing (Lang & Bradley, 2010; Schupp et al., 2006; Stockburger et al., 2008, 2009; Weymar et al., 2012). Pictures of high-calorie food induced larger LPC amplitudes in the time interval from 350–550 ms when participants were instructed to think about the long-term consequence of eating them (Meule et al., 2013). Moreover, food pictures yielded larger LPC amplitudes compared to non-food items (Nijs et al., 2008). Overall, relative to non-food items, food stimuli seem to induce attentional priority, followed by an assessment of action capacities that systematically depend on certain food features (Nijs et al., 2010).

Another variable affecting food choice is food deprivation, an important physiological state that plays a key role in motivating individuals to seek food (e.g., Piech et al., 2009; Anselme & Güntürkün, 2019). Food deprivation influences the immediate valence of food stimuli as well as spontaneous motivational tendencies (Seibt et al., 2007). Food deprivation has been reported to increase the LPC amplitude in the ERP between 450 and 600 ms following stimulus onset (Stockburger et al., 2009), which might be interpreted as an adjustment of perceived action capacities, induced by a highly motivated state. But again, the evidence for such adjusted perception of action capacities was only indirect. A recent study found inconsistent evidence for overt action preparation during food perception when comparing touchscreen-based and joystick-based methods, thus suggesting to use indirect measures of action preparation (Meule et al., 2019).

In summary, previous research has consistently shown effects of food features on selective attention and eating motivation and has identified the time course of their evaluation. A similar bi-phasic time course has been documented for object perception, with attention followed by motor preparation. However, details of the relationship between food features and action tendencies are less clear. Specifically, we do not know whether the introduction of food stimuli changes people’s assessment of their subjective representation of peripersonal space and how such changes might depend on specific food features, such as calorie content or valence and the participants’ satiation/hunger state. Therefore, in the present study two reachability assessments for food items were performed. In Experiment 1 we investigated whether and how peripersonal space for food items depends on participants’ evaluation of food objects: How are reachability judgments influenced by the items’ valence and calorie content and by the satiation status of participants? We hypothesized that being hungry influences the participants’ visual perception of their peripersonal space, yielding a change in the reachability judgements for high-calorie and high valence food pictures which would increase the expanse of the subjective representation of participants’ peripersonal space. This would strengthen the interpretation of food evaluations in terms of action tendencies. Our prediction was, such increase of the expanse of subjective representation of peripersonal space would be affected by food deprivation and similarly by the characteristics of food items, that is, by high valence and high calories.

In Experiment 2 we aimed to replicate the results of Experiment 1 and to exploit the temporal resolution of ERP components in hungry vs. satiated conditions, in order to obtain information about the time course of the observed effects and how they depend on the spatial location of the food pictures. Specifically, we studied whether and when during food exposure there is attentional enhancement directed at food within reach and how it depends on food properties (calories, valence) and deprivation/satiation status. We expected a biphasic pattern with early attentional enhancement (P1 component) and later motivational signatures of action preparation (LPC).

## Experiment 1

### Methods

Participants: Sixteen omnivorous normal-weight individuals were initially recruited for the study via posters, flyers, email, and social networks. One participant had to be excluded from the analyses due to inconsistent reachability ratings that suggest misunderstanding of instructions. The remaining 15 participants (all right-handed, 6 females; *mean BMI* = 23.61 kg/m^2^, *SD* = 2.21) were between 21 and 34 years of age (*M* = 27.6, *SD* = 4.01) with normal or corrected-to-normal vision. Post-hoc determination of power revealed a 70 % chance of correctly rejecting the null hypothesis of no significant effect for with a total of 15 participants, given an expected medium effect size and p < .05. The experiment was approved by the ethics committee of the University of Potsdam (Reference number 76/2016). Participants gave their informed consent prior to their inclusion in the experiment.

Stimuli and materials: Food pictures were taken from the database provided by Blechert et al. (2014). Sixty stimuli were selected based on the calorie content of the depicted food (high vs. low-calories) and valence ratings (high vs. neutral valence) provided by the database. According to Wang et al., (2018) the valence of food is associated with its tastiness (whether it is appetitive or aversive), which specifies its hedonic value and elicits the execution of selection behaviors. The valence dimension was included here following up on previous literature, according to which “…*the studies on nutritiousness effects are inconclusive with regard to affective aspects, as high calorie content is not equivalent to positive valence*” (Schacht et al., 2016, p. 34).

**Figure 1:**
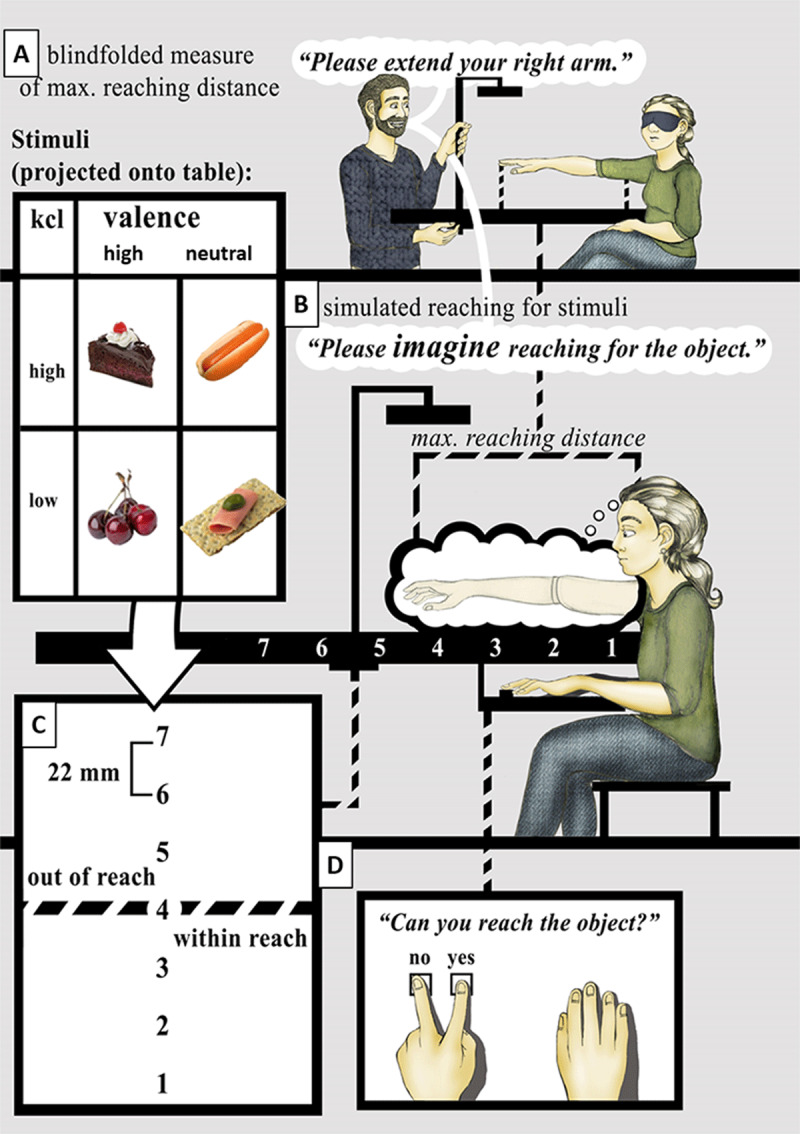
Experimental procedure. **A)** Participants extended their right arm (blindfolded) to establish their peripersonal space. **B)** They were reminded to keep this posture in mind when making their reachability judgments. **C)** Stimuli from different categories were presented at seven distinct positions; position four was aligned with the maximum extension of the right hand. **D)** Yes or no responses were performed by the left hand while the right hand remained stationary next to the keyboard below the table.

All stimuli were displayed by a mini projector (Acer C110 WXGA 50 Lumens LED) projecting the pictures on a table (see ***Figure 1***). In total, there were four stimulus categories: 30 pictures of food with high-calorie content (15 each of high vs. neutral valence) and 30 of low-calorie content (15 each of high vs. neutral valence). Overall, the average total calorie content of high and low-calorie items was 490.39 kcal, *SD* = 161.96 vs. 83.49 kcal, *SD* = 48.88; *t*(58) = 13.174, *p* < .001. High and neutral valence pictures obtained mean ratings of 59.73 (*SD* = 6.16) vs. 42.39 (*SD* = 5.37), *t*(58) = 11.615, *p* < .001 (see ***Table 1***). As revealed by analyses of variance (ANOVA) with the factors calorie content and valence, the four picture categories did not differ in object size (proportion of non-white pixels relative to total number of pixels), *F*(3, 56) = 0.177, *p* = .912, brightness (luminance of all non-white pixels of the gray scale image and the white background, *F*(3, 56) = 2.103, *p* = .112, contrast (SD of luminance across all non-white pixels of the gray-scaled image), *F*(3, 56) = 1.060, *p* = .373), or complexity (for definition please see Blechert et al., 2014), *F*(3, 56) = 0.457, *p* = .714. The images were edited to be projected at different locations along the mid-sagittal line and showed immediately edible food items (no raw food except for sushi) and appeared easy to grasp (Linkenauger et al., 2009). Images were oriented with the food item’s main axis perpendicular to the observer’s mid-sagittal plane and there was no blank space between the lower border of a photo and the lower border of a food item.

**Table 1:** Stimulus characteristics taken from Blechert et al.’s (2014) database.


FOOD CATEGORIES	TOTAL CALORIES	VALENCE RATINGS	OBJECT SIZE	BRIGHTNESS	CONTRAST	COMPLEXITY

High Calorie/High Valence	490.52	56.51	.325	37.52	56.61	.087

High Calorie/Neutral Valence	490.26	41.04	.326	36.48	50.82	.082

Low Calorie/High Valence	71.47	62.95	.311	34.29	49.13	.077

Low Calorie/Neutral Valence	95.52	43.74	.311	29.43	53.77	.088


*Note*: Total calorie values expressed in Kcal, valence ratings from 1 (very low) to 100 (very high), object size (the proportion of non-white pixels relative to total number of pixels), brightness (the luminance of all non-white pixels of the grey-scale image and the white background), contrast (the SD of luminance across all non-white pixels of the gray-scale image), and complexity (for definition please see Blechert et al., 2014).

Design and procedure: The experiment was divided into two sessions. Both sessions took place one to five days apart but always between 11:30 am and 2:00 pm to control for circadian effects. For the “hungry condition” session, participants were asked to abstain from eating for about three hours prior to the experiment. Before the “satiated condition” session, participants were invited to eat *ad libitum* until satiated at the university’s cafeteria (Mensa), followed immediately by the experiment. The order of the hungry and satiated sessions was counterbalanced across participants (within-subject design). At the beginning of the first session, participants signed the consent form and completed a hunger scale (described below). The same scale was completed at the beginning of the second session. Measures of reachability judgments were treated as the dependent variable and Condition (hungry and satiated), Calorie (high and low) and Valence (high and neutral) as the independent variables. In order to assess the effect of the independent variables on reachability judgments, repeated measures analyses of variance (ANOVA) were applied.

During the experiment, the participant was seated at the small edge of a table (approximately 60 × 105 cm; white surface) onto which the food pictures were beamed. The table was height-adjusted to align with the participant’s lower part of the sternum to minimize their range of movement. Before the experiment, each participant was blindfolded and asked to maximally extend their right arm and hand while touching the surface of the table to define their objective individual peripersonal space. The tip of the middle finger (***Figure 1A***) was taken as the “standard stimulus” reference. The participant subsequently was reminded to keep this posture in mind when making reachability judgments (***Figure 1B***). All stimuli were presented at seven distinct positions: One being aligned with the maximum extension of the right hand, that is, the “standard stimulus” position, three nearer positions, and three positions farther away, at intervals of 2.2 cm, as measured from the lower edge of the stimulus image frame (***Figure 1C***). During the experiment, each stimulus appeared repeatedly at each of the selected positions (method of constant stimuli) and remained in the projected position until participants decided whether it was reachable or not. Decisions were made by pressing one of two keys on a keyboard (yes and no keys) hidden from sight below the table. Keys were pressed with the left hand while the right hand remained stationary next to the keyboard (***Figure 1D***). After the response, a blank screen appeared for 1500 ms and the next stimulus was presented. The order of presentation was randomized across all stimuli and positions. The combination of 60 stimuli × 7 positions yielded 420 trials for each participant, taking about 20 min to complete.

Data collection and data analysis: Data were collected using E-Prime software (v.1.2.). The individual limit of peripersonal space was determined by probit analysis (the inverse of the cumulative standard normal distribution function) of the response proportions in SPSS Statistics 24.0 (*IBM Knowledge Center*, 2014). This method yielded subjective reachability limits, the point of subjective equality (PSE), which was calculated by computing the value that corresponds to 50% “yes” responses.

The difference between the PSE and the standard stimulus position is called the constant error (CE) (Postman & Bruner, 1946) and expresses the subjective reachability perception in comparison to the maximal extension of the arm. A positive CE indicates that the point of subjective reachability is positioned further away than the standard stimulus position. Thus, subjective space is expanded relative to objective peripersonal space. Conversely, a negative CE value indicates a smaller subjective than objective reachability estimate. The CE values were statistically analyzed using the Software SPSS Statistics 24.0 by submitting them to a three-way repeated-measures ANOVA with factors condition (hungry vs. satiated), calorie content (high vs. low), and valence (high vs. neutral). Variances of the differences between levels of the factors were assumed to be equal (null hypothesis). Post-hoc *t*-tests were calculated to follow up on interaction effects. The variability of any pair’s comparison was assumed to be normally distributed. If the p-value was less than .05, the null hypothesis was rejected and concluded that a significant difference exists. Alpha levels were adjusted with the Bonferroni procedure in order to reduce Type I errors. For effects involving repeated measures, the Greenhouse–Geisser procedure was used to correct for violations of sphericity. To support the ANOVA results we also used mixed-effects logistic regression analysis (Pinheiro & Bates, 2000). To do that, we included “condition” and “position” as fixed effects and the response for each trial (“reached or not reached”) as predicted variable. We considered that each food item appeared for each participant and each participant might judge each item different from the other one. This means that variation between participants and items might exist. In addition, we also contemplated that the effect of condition (hungry – satiated) could change across participants. Thus, we included by-participants intercepts and random slopes for the effect of condition and items as random intercepts.^1^

#### Other Measurements

##### Subjective food preference ratings

In order to compare the reachability results with subjective food preferences, we administered a post-experimental questionnaire. Participants rated all food pictures, randomized in order, in response to the question “How pleasant would it be for you to eat the presented food now?” on a five-point scale (one = not so much; five = very much). The ratings were submitted to a three-way repeated-measures ANOVA with factors condition (hungry vs. satiated), calorie content (high vs. low), and valence (high vs. neutral). With the aim to back up the results due to the variation between participants and items, and to draw conclusions about “food” in general, rather than about the specific items presented, a linear mixed model was performed.^1^ We included condition, calorie content and valence as fixed effects and rating scores were used as predicted variable. We also included “condition” as random slopes and participants and items as random effects.

##### Hunger ratings

Hunger ratings were obtained by means of a visual analogue scale (VAS) (Blundell et al., 2010), using a 100 mm horizontal line presented on paper accompanied with the printed instruction “Please indicate how hungry you feel right now by making a vertical line on the scale at the appropriate point.” The left end of the line was anchored with the words “not at all”, and the right end was anchored with the word “extremely”.

### Results and Discussion

#### Hunger Ratings

Subjective ratings of hunger were enhanced during the hungry condition (*M* = 82.7, *SD* = 5.93) as compared to the satiated condition (*M* = 15.3, *SD* = 8.12), *t*(14) = 34.643, *p* < .001.

#### Reachability task

**Figure 2:**
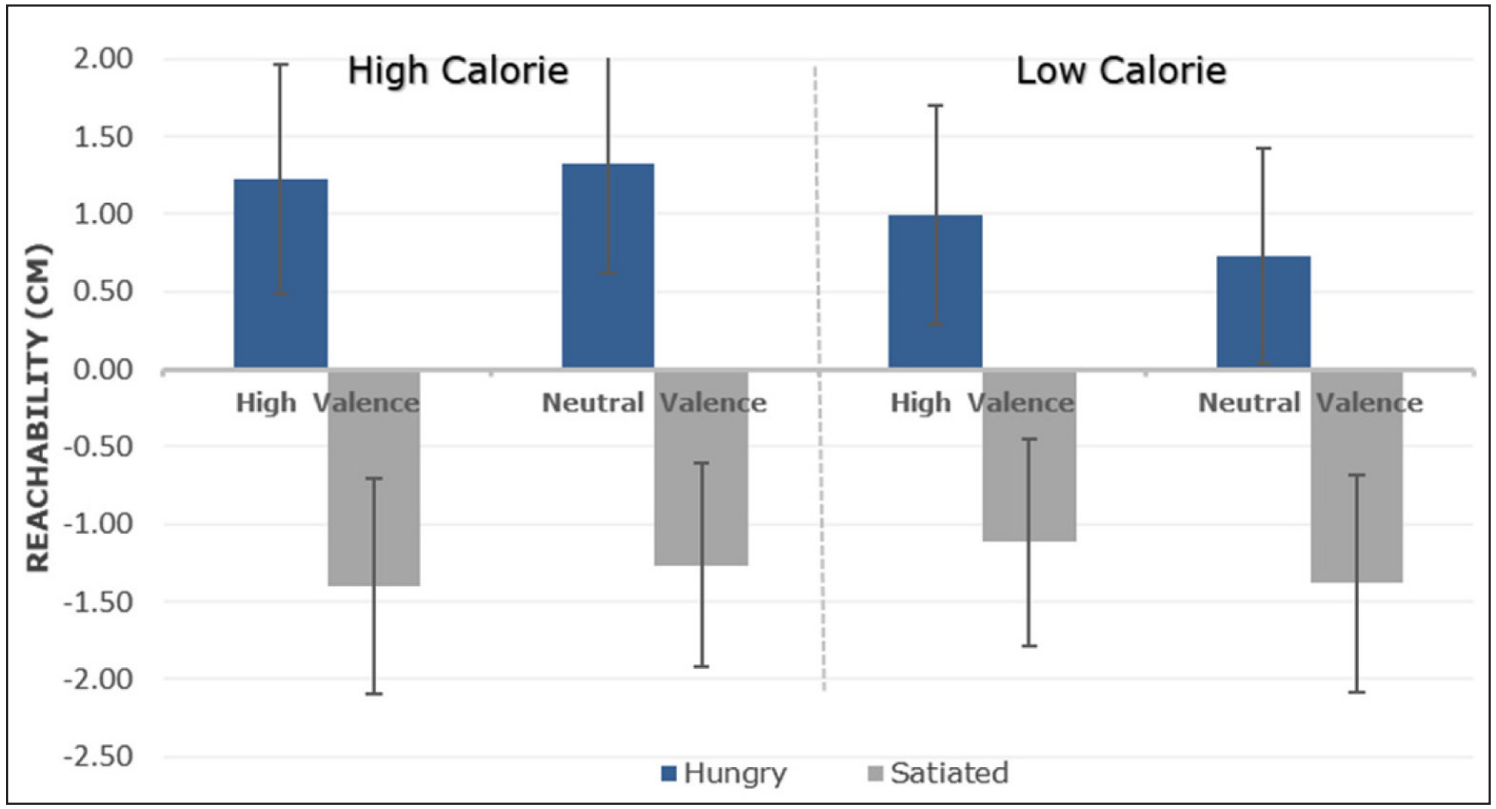
Mean scores of the perception of subjective reachability limit for each food category (calorie and valence) as a function of hungry and satiated. 0 (zero) indicates the true value of the maximal extension of the arm. Positive values indicate that the limits of reachability were further out, that is, peripersonal space was increased. Standard error (SE) shown as vertical lines.

The standard stimulus reachability boundary (maximal hand extension) was located at 70.53 cm (*SD* = 6.27 cm). The difference between the PSE and this standard stimulus (zero value) represents the CE (constant error) and is visualized in ***Figure 2***. In the hungry condition the CE was +1.06 cm while in the satiated condition, the CE was –1.29 cm. This difference was significant, *F*(1,14) = 28.263, *p* < .001, *η*_*p*_*^2^* = .669, but Condition also interacted with Calories, *F*(1,14) = 12.509, *p* = .003, *η*_*p*_*^2^* = .472. A follow-up test showed that the Calorie effect on reachability judgments was only present in the hungry state, *t*(14) = 3.550, *p* = .003 (Bonferroni corrected), but not in the satiated state, *t*(14) = –0. 617 *p* = .547. ***Figure 2*** indicates that this interaction was due to a larger increase of reachability for high than low-calorie items. No other effects or interactions were observed, all *p* values > .05. Moreover, we fitted a logistic mixed model to predict the participant’s response by condition (hungry and satiated) and position (1 to 7) as predictors. The model also included by-participants intercepts and random slopes for the effect of condition and items as random intercepts. The model’s intercept was –0.05 (*SE* = 0.51) which corresponds to the mean value of the participants’ responses. Within this model, the interaction of condition and position was significant (Beta = 0.16, *SE* = 0.07, *p* = .016), indicating that the selection of a reachable position interacts with the satiation state of the participants, backing up the main effect of condition in the ANOVA analysis with the CE index.

Results of the reachability task suggest that the introduction of food modifies the estimation of peripersonal space, affecting the reachability judgments. Thus, visual perception and motor simulation seems to be influenced by hunger and are differentially affected by the stimulus dimension of calorie content. Hunger increases peripersonal space relative to the satiated state by about two centimeters. This increase of peripersonal space by hunger is more pronounced for high-calorie items. This interaction suggests the presence of an incentive salience, which may have been translated into intention. In other words, hungry participants may be more motivated to perform a goal-directed action to reach, and therefore physically interact with the perceived high-calorie food items. However, these behavioral results cannot determine whether high-calorie items were indeed attended more intensely than low-calorie items. Moreover, we have no information about the time course of participants’ responses because they were instructed to emphasize judgment accuracy. To obtain this information, a second experiment was performed, reported after the next section.

#### Subjective food preference ratings

Food stimuli were rated as more pleasant when participants were hungry rather than satiated (hungry: *M* = 3.37, *SD* = 0.83; satiated: *M* = 2.21, *SD* = 0.75); *F*(1,14) = 19.724, *p* = .001, *η*_*p*_*^2^* = .585. The main effect of valence was also reliable, with larger values for high- than neutral-valence food items (high: *M* = 2.98, *SD* = 0.85; neutral: *M* = 2.59, *SD* = 0.72), *F*(1, 14) = 17.624, *p* = .001, *η*_*p*_*^2^* = .557. Also, an interaction Condition × Calorie × Valence was found, *F*(1, 14) = 18.770, *p* = .001, *η*_*p*_*^2^* = .573. To disentangle this interaction, we conducted separate repeated measures ANOVAs on factors Calorie and Valence for each condition. In the hungry condition there was a Calorie × Valence interaction, *F*(1,14) = 12.157, *p* < .004, *η*_*p*_*^2^* = .465. A follow-up test showed a main effect of high vs. neutral valence for the low-calorie items *t*(14) = 4.163, *p* = .001 (Bonferroni corrected). No other effects or interactions were observed, all *p* values > .05. Moreover, a linear mixed model was fitted to predict the participant’s ratings by condition (hungry vs. satiated), calorie (high vs low) and valence (high vs. neutral). The model’s intercept was 2.79 (*SE* = 0.12) and represents the mean of the Rating scores. Within this model, there was a significant effect of condition (Beta = 1.16, *SE* = 0.29, *p* < .001) and valence (Beta = 0.40, *SE* = 0.09, *p* < .001), backing up the main effect of condition and valence in the ANOVA analysis with the Ratings scores.

Overall, there was a general preference for high valence over neutral food, confirming the validity of item selection. Moreover, participants reported higher ratings in the hungry than in the satiated condition, suggesting that food is subjectively perceived as more pleasant before than after eating.

## Experiment 2

This experiment aimed to replicate the behavioral findings of Experiment 1 and to shed light on the neurocognitive processes (e.g., attentional and motivational) underlying the effects of hunger and calorie on spatial food perception.

### Methods

Participants: Twenty-seven normal weight participants were recruited through a web-based application. Five participants (3 females) had to be excluded from ERP analyses due to technical problems or poor EEG quality. The remaining 22 participants (18 females) were 20–38 years of age (*M* = 26.31, *SD* = 4.62). No participant was currently on a diet or reported any food allergies, chronic disease, or history of eating disorder. Post-hoc determination of power revealed 89 % chance of correctly rejecting the null hypothesis of no significant effect for with a total of 22 participants, given an expected medium effect size and *p* < .05.

All participants reported normal or corrected-to-normal visual acuity. According to the Edinburgh handedness questionnaire (Oldfield, 1971), all participants were right-handed (score = 85.22). The experiment was approved by the ethics committee of the University of Potsdam (Reference number 76/2016). Participants gave their informed consent prior to the experiment.

Stimuli and materials: The stimuli and apparatus were the same as in Experiment 1, except that the projector was a Philips Pico Pix 3414 (WVGA – 100 ANSI-Lumen-Wit).

Design and procedure: The experiment consisted of two sessions: hungry and satiated condition, both being conducted between 11:30 am and 2:00 pm, and counterbalanced in order (as in Experiment 1). The second session took place one to five days after the first session. At the beginning of the first session participants signed the consent form and proceeded to complete the handedness questionnaire and the VAS, used in the first experiment. Variables were the same as in Experiment 1. In addition, in the ERP analysis, the independent variable distance (near and far) was included (see below), and measures of P1 and LPC amplitude as dependent variables.

The experimental task was the same as in Experiment 1, except that the stimuli were presented for 2000 ms; during this time participants had to decide whether or not they could reach the stimulus and brain activity was recorded. After the termination of stimulus presentation, the screen went blank for 1800 to 2200 ms to indicate the response interval. Trials in which participants pressed more than once and gave inconsistent responses (yes and no) were not considered for analysis.

#### Data collection

##### Reachability task

Experiment 2 was controlled by *Presentation® Software* (“Neurobehavioral Systems,” Inc).

**Figure 3:**
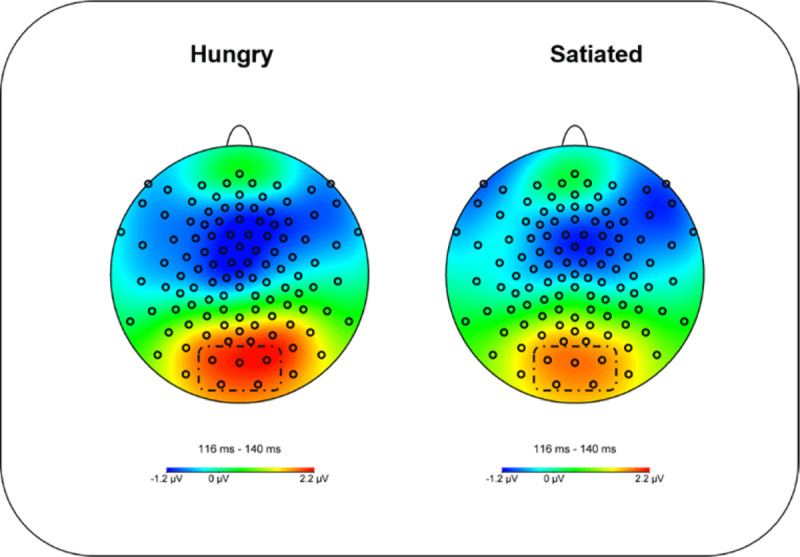
Topography of maximal amplitudes of the P1 component between 116 and 140 ms in hungry and satiated conditions. Marked are the electrodes of the P1 ROI (70,74,75,82,83).

*Event-Related Brain Potentials (ERPs).* Electrophysiological data were collected from a 129-lead geodesic sensor net (Electrical Geodesics, Inc. [EGI]) at a sampling rate of 250 Hz, with the sensor at the vertex as reference electrode. Impedances were kept below 30 kOhm, as recommended by manufacturer guidelines. Ocular blink artifacts were eliminated using the surrogate model of multiple source eye correction (Berg & Scherg, 1994), included in BESA software version 6.0 (BESA GmbH). Analysis was performed using Brain Vision Analyzer (BVA), version 2.1. Activity at the (Cz) reference electrode was retrieved (sign-inversed average that reconstructs the original reference activity). The EEG was high-pass filtered at 0.1 Hz, as recommended by Tanner et al. (2015), and low-pass filtered at 30 Hz, including a 50 Hz notch filter. Data from bad channels were interpolated participant-wise. Artefacts due to horizontal and vertical eye movements, cardiac pulses, muscular activity and other artifacts were removed using independent component analysis, specifically the fast ICA Algorithm, implemented in BVA (“BrainVision Analyzer Support Tip – ICA Demystified,” 2014). Then, EEG data were segmented into epochs extending from –200 until +1000 ms relative to stimulus onset. After correcting for a 200-ms pre-stimulus baseline, epochs were discarded as artefact-contaminated when any amplitude exceeded +/–200 microvolt (μV). Overall number of valid segments was high (> 90%). Data was averaged separately according to the category combinations calories (high vs. low), valence (high vs. neutral) and stimulus position. For the stimulus positions, ERPs were merged into two contrasting categories: “Near” (position one, two and three) and “Far” (position five, six and seven) to obtain a balanced distribution. As a measure of selective attention and sustained elaborative processing we focused on the P1 and LPC components (e.g., Schacht et al., 2016; Stockburger et al., 2009). In accordance with Schacht et al. (2016), the P1 was analyzed in the occipital area for the time segment 116–140 ms. Based on Meule et al. (2013) LPC activity was measured at parieto-occipital electrodes between 350 and 550 ms,

The P1 was averaged and analyzed across the occipital electrodes of the geodesic sensor net (70, 74, 75, 82, 83) where its amplitude was maximal in both conditions (hungry and satiated). Based on Schacht et al. (2016), these electrodes were collapsed and analyzed in the time segment of 116–140 ms (see ***Figure 3***).

**Figure 4:**
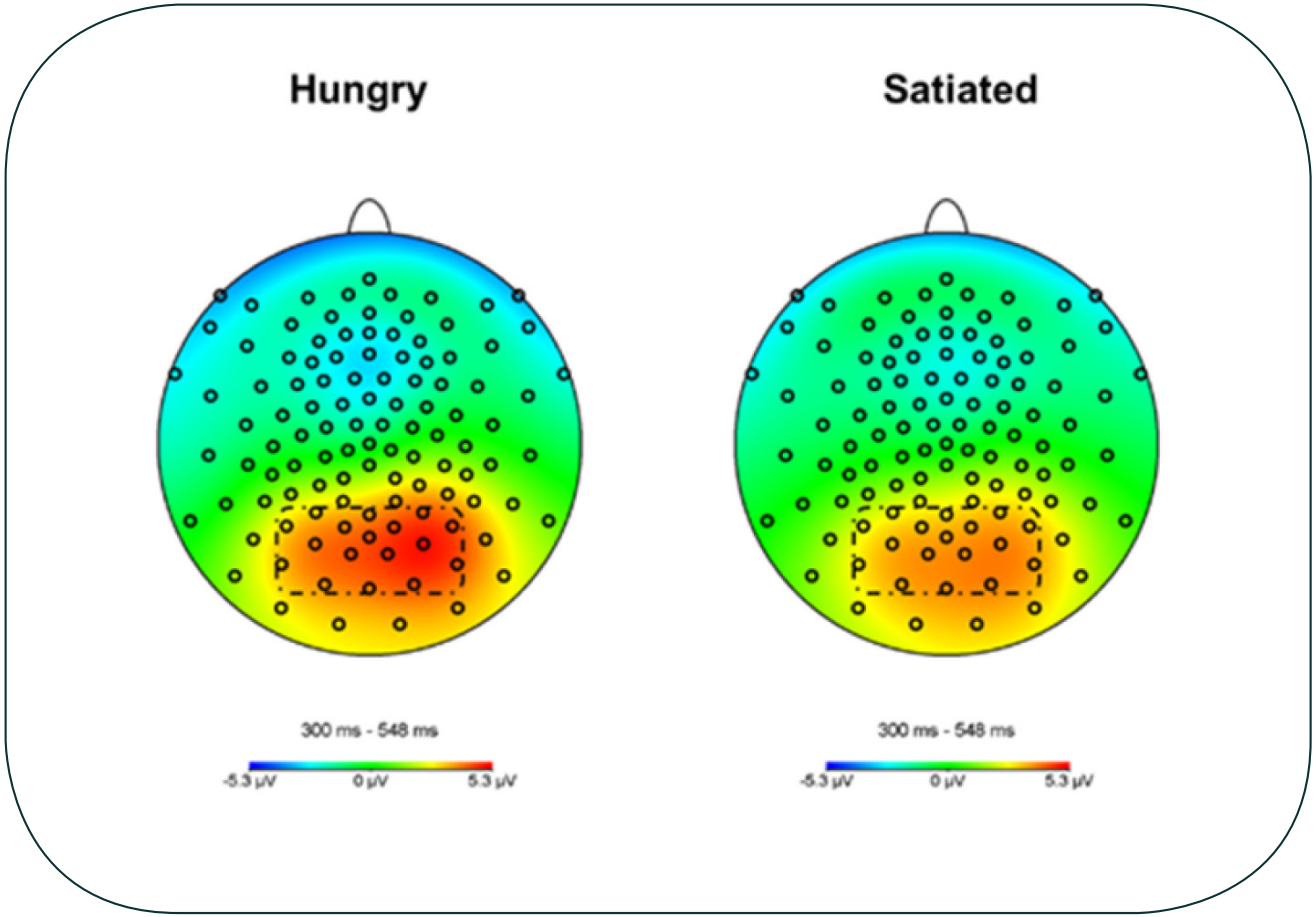
Topography of maximal amplitudes of the Late Positive Component between 300 and 548 ms in hungry and satiated conditions; marked are the electrodes used for data analysis (59-60-62-65-66-67-70-71-72-75-76-77-83-84-85-90-91).

The maximal activity of the LPC was located at the parieto-occipital scalp in the time window between 300–548 ms, for the electrode selection a symmetrical cluster (right and left hemisphere) in the area of main activity was considered (59-60-62-65-66-67-70-71-72-75-76-77-83-84-85-90-91); The EEG from these electrodes was collapsed and used for statistical analysis (see ***Figure 4***).

Mean amplitudes of P1 and LPC were submitted to 4-way repeated measure ANOVAs with factors Condition (hungry vs. satiated), Distance (near vs. far), Calories (high vs. low), and Valence (high vs. neutral). Interactions were followed up with post-hoc *t*-tests using Bonferroni correction. For effects involving repeated measures, the Greenhouse–Geisser procedure was used to correct for violations of sphericity. Moreover, to support the ANOVA results, as in the other tasks, we used mixed-effects analysis.

#### Other Measurements

Subjective food preference and hunger ratings were performed as in Experiment 1.

### Results and Discussion

#### Hunger Ratings

Subjective ratings of hunger were enhanced during the hungry condition (*M* = 82.05, *SD* = 9.71) as compared to the satiated condition (*M* = 8.41, *SD* = 8.07), *t*(21) = 30.409, *p* < .001.

#### Reachability task

**Figure 5:**
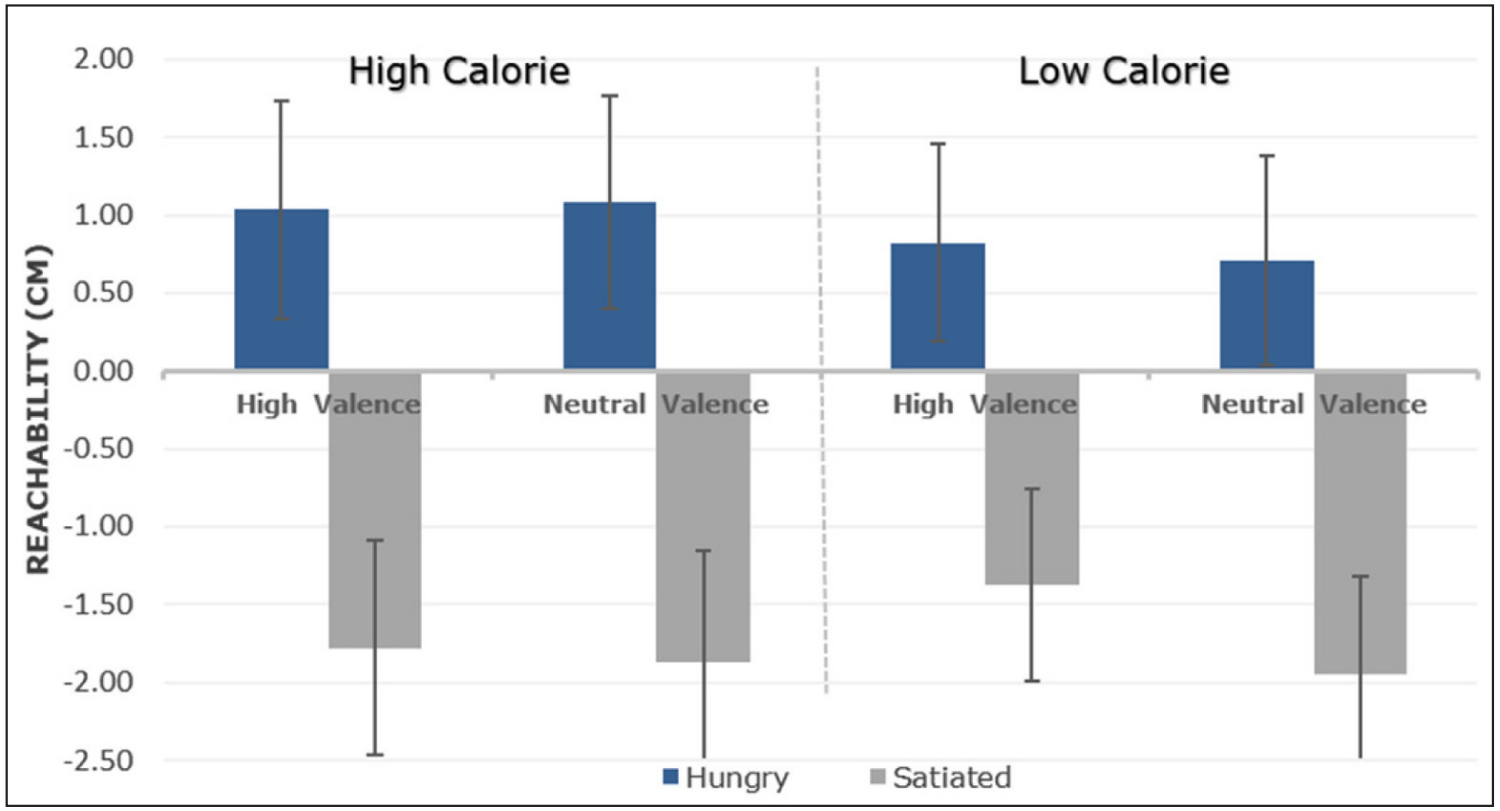
Mean subjective reachability limits for each food category (calorie and valence) as a function of hungry and satiated. Conventions as in Figure 2.

In the hungry condition the estimation of the CE was +0.91 cm, whereas in the satiated condition, the CE was –1.74 cm (see ***Figure 5***), *F*(1,21) = 22.425, *p* < .001, *η*_*p*_*^2^* = .516. Condition interacted with calories, *F*(1,21) = 10.010, *p* = .005, *η*_*p*_*^2^* = .323. Follow-up analysis showed a main effect in the hungry condition for high-calorie in comparison with low-calorie food items, *t*(21) = 2.757, *p* = .012 (Bonferroni corrected). No such calorie effect was observed when participants were satiated, *t*(21) = –0.873, *p* = .392. No other effects or interactions were observed, all p values > .05. Moreover, we fitted a logistic mixed model to predict the participant’s response by condition (hungry vs. satiated) and position (1 to 7) as predictors. The model also included condition as random slopes, participants and items as random effects. The model’s intercept was –0.20 (*SE* = 0.36) which corresponds to the mean value of the participants’ responses. Within this model, the interaction of condition and position was significant (Beta = –0.25, *SE* = 0.05, *p* < .001), indicating that the selection of a reachable position interacts with the satiation state of the participants, backing up the main effect of condition in the ANOVA analysis with the CE index.

Reachability results in Experiment 2 replicated those of Experiment 1 where food stimuli were perceived as being more reachable when participants were hungry, especially for high-calorie items. The effect size was also similar to Experiment 1, with around 2.5 cm. This increases confidence in the observed behavioral effect and justifies its further exploration with ERPs, as described next.

#### ERP measurements

**Figure 6:**
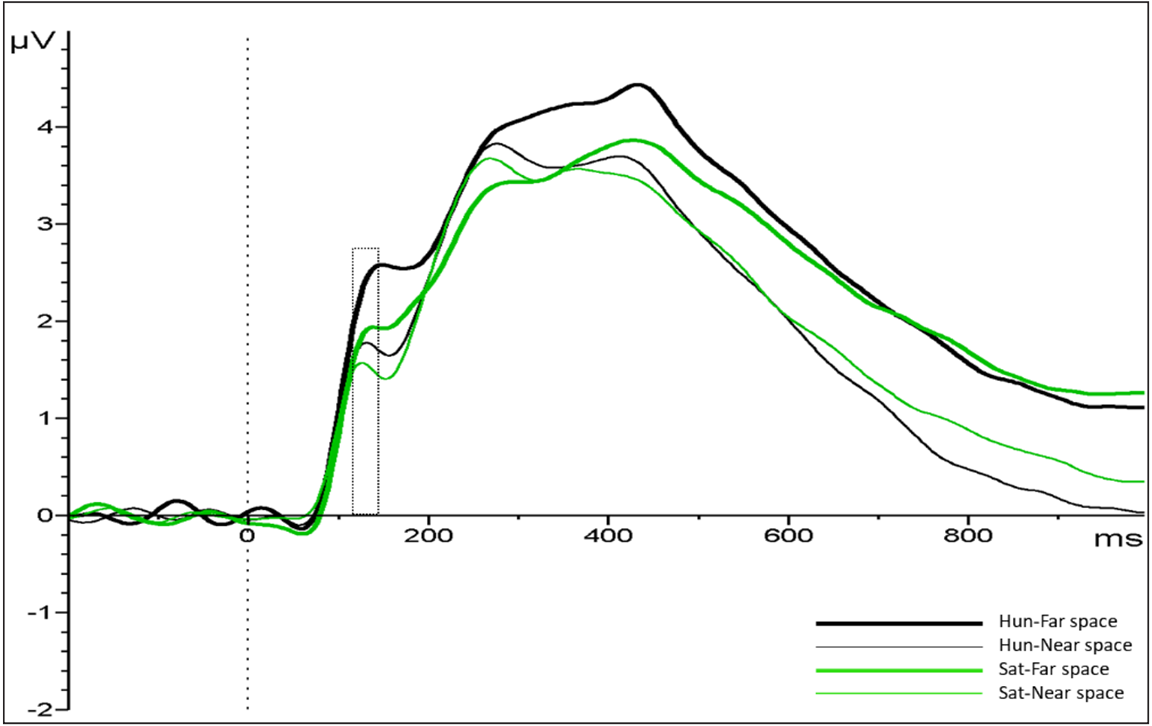
ERP waveforms, pooled within the P1 ROI, in hungry and satiated condition in far and near space. Marked is the interval of the P1 amplitude analysis.

Analyses of the P1 (116–140 ms) presented a main effect of Distance, showing larger amplitudes for the far in comparison with the near space, (*M* = 2.09, *SD* = 1.49; vs. *M* = 1.66, *SD* = 1.58); *F*(1, 21) = 8.416 *p* = .009, *η*_*p*_*^2^* = .286. (see ***Figure 6***). Moreover, a significant Condition × Distance interaction was found, *F*(1,21) = 4.391, *p* = .048, *η*_*p*_*^2^* = .173. Post-hoc analysis showed a larger P1 amplitude for far space items vs. near space when participants were hungry (*M* = 2.31, *SD* = 1.58 vs. *M* = 1.73, *SD* = 1.66); *t*(21) = 3.611, *p* = .002 (Bonferroni corrected). In contrast, no location differences were observed in the satiated condition (*M* = 1.87 *SD* = 1.40 vs. *M* = 1.59, *SD* = 1.48); *t*(21) = 1.596, *p* = .125 (see ***Figure 6***). No other main effects or interactions were significant, all *p* > .05. Furthermore, to support the ANOVA results, we fitted a linear mixed model. For that purpose, we included “condition” and “distance” as fixed effects and the amplitude for each trial (µV) as predicted variable. We also included by-participants intercepts and random slopes for the effect of condition and items as random intercepts. The model’s intercept was 1.87 (*SE* = 0.27) and represents the mean value of the P1 amplitude (µV). Within this model, the interaction Condition*Distance was not significant (Beta = 0.29, *SE* = 0.16, *p* = .078). However, there was a significant effect of distance (Beta = 0.44, *SE* = 0.08, *p* < .001), backing up the main effect of distance in the ANOVA used for the analysis of the amplitudes values (µV).

**Figure 7:**
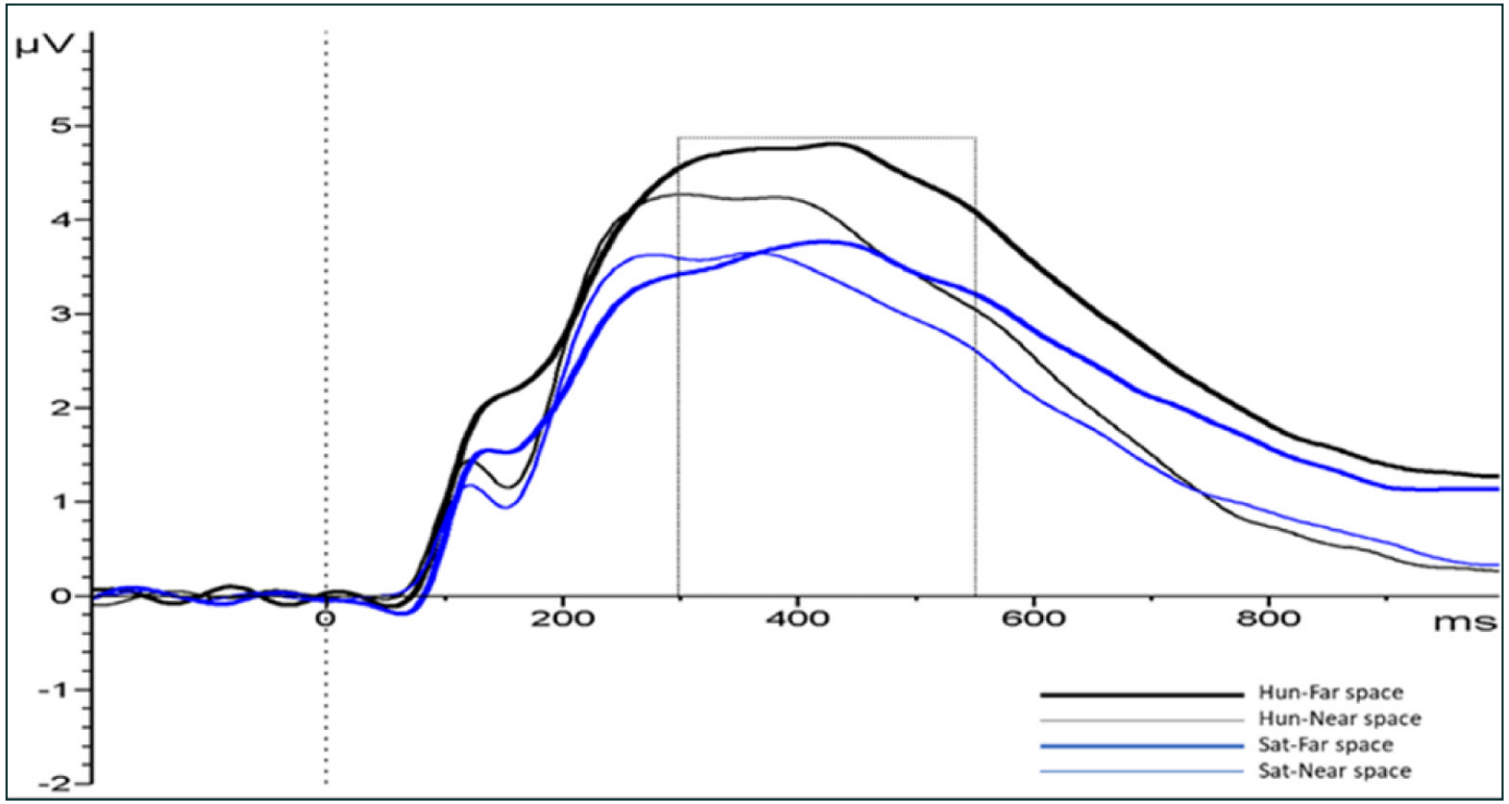
Mean amplitude of LPC in hungry and satiated condition in far and near space. Pooled ERPs electrodes from the parieto-occipital area (59-60-62-65-66-67-70-71-72-75-76-77-83-84-85-90-91). Marked is the interval of the LPC amplitude analysis.

LPC analyses in the time window 300–548 ms showed a main effect of Condition (hunger vs. satiated), (*M* = 4.24, *SD* = 2.37; vs. *M* = 3.45, *SD* = 1.79); *F*(1, 21) = 7.094, *p* = .015, *η*_*p*_*^2^* = .253, replicating prior findings (see Stockburger et al., 2009). The factor Distance showed a significant main effect, *F*(1,21) = 18.308 *p* < .001, *η*_*p*_*^2^* = .466, and interacted with condition *F*(1, 21) = 8.663, *p* = .008 *η*_*p*_*^2^* = .292. Follow-up analysis showed that in the hungry condition, food stimuli in far positions prompted larger LPC amplitudes (see ***Figure 7***) than at near positions (*M* = 4.60, *SD* = 2.38 vs. *M* = 3.89, *SD* = 2.16), *t*(21) = 5.114, *p* < .001 (Bonferroni corrected). No such location effects were observed when participants were satiated *M* = 3.56, *SD* = 1.71 vs. *M* = 3.34, *SD* = 1.60), *t*(21) = 1.603, *p* = .124. No other effects or interactions were observed, all p values > .05. As in the P1 amplitude analysis, we fitted a linear mixed model. We included “condition” and “distance” as fixed effects and the amplitude for each trial (µV) as predicted variable. We also included “condition” as random slopes and participants and items as random effects. The model’s intercept was at 3.84 (*SE* = 0.39) and represents the mean value of the LPC amplitude (µV). Within this model, the interaction Condition*Distance was significant (Beta = 0.48, *SE* = 0.16, *p* = 0.004). Moreover, there was a significant effect of condition (Beta = 0.80, *SE* = 0.30, *p* = .014) and distance (Beta = 0.49, *SE* = 0.08, *p* < .001), backing up the main effect of condition and distance in the ANOVA analysis of the ERP amplitudes.

In sum, Experiment 2 obtained ERP components while participants evaluated the reachability of food items while either being hungry or satiated. This allowed us to study the time course of such food evaluations and revealed an attentional enhancement, reflected in early and late components. The P1 component indicated that more attention was deployed towards more distant than nearby stimuli. The late-emerging LPC showed that food items in far space received more motivated attention when participants were hungry and when stimuli were far away.

#### Subjective food preference ratings

Ratings from one participant were missing. Food stimuli were rated as more pleasant when participants were hungry rather than satiated (*M* = 3.30, *SD* = 0.62 vs. *M* = 1.61, *SD* = 0.72), *F*(1, 20) = 177.250, *p* < .001, *η*_*p*_*^2^* = .899. Pleasantness ratings were higher for high valence than neutral food stimuli, as indicated by the significant main effect of Valence (*M* = 2.66, *SD*= 0.72 vs. *M* = 2.26, *SD* = 0.62), *F*(1, 20) = 49.708, *p* < .001, *η*_*p*_*^2^* = .713. An interaction between Calorie x Condition, *F*(1, 20) = 33.699, *p* < .001, *η*_*p*_*^2^* = .628 was also found. Follow-up comparisons showed that hunger increased the pleasantness of high compared to low-calorie items, *M* = 3.44 *SD* = 0.51, vs. *M* = 3.18, *SD* = 0.55; *t*(20) = 3.083, *p* = .006 (Bonferroni corrected). In contrast, being satiated decreased the effect of high- vs. low-calorie items, *M* = 1.46, *SD* = 0.68 vs. *M* = 1.76, *SD* = 0.71; *t*(20) = –5.375, *p* < .001 (Bonferroni corrected). This effect indicates that hungry participants prefer more high-calorie content items and this effect is reversed when satiated. Moreover, an interaction Calorie x Valence was found, *F*(1, 20) = 5.326, *p* < .032, *η*_*p*_*^2^* = .210. Post-hoc analyses reflected significant differences (*p* < .05) for all high-valence items in comparison with neutral ones (Bonferroni corrected).

Furthermore, a linear mixed model was fitted to predict the participant’s rating by condition (hungry vs. satiated), calorie (high vs low) and valence (high vs. neutral). The model’s intercept was 2.46 (*SE* = 0.12) and represents the mean of the Rating scores. Within this model, there was a significant effect of condition (Beta = 1.70, *SE* = 0.14, *p* < .001) and valence (Beta = 0.39, *SE* = 0.08, *p* < .001), supporting the main effect of condition and valence in the ANOVA of the Ratings scores.

## General Discussion

The first aim of the present study was to determine whether perceived peripersonal space for food items is affected by hunger vs. satiation states and by specific food characteristics (valence and calorie). Moreover, in the second experiment, we intended to replicate the behavioral results of the first experiment and to elucidate its time course. We therefore recorded ERPs with the purpose to functionally localize the obtained effects in terms of the related neurocognitive processes and their temporal unfolding. Finally, a questionnaire was administered in order to investigate subjective preferences for the food pictures and their susceptibility to motivational states.

### Reachability task

We found that participants’ perception of peripersonal space, measured by the point of subjective equality (PSE) in a reachability judgment task, was wider in the hungry condition when compared to the standard stimulus (i.e., the maximal extension of the right hand). In contrast, perceived peripersonal space was narrowed in the satiated condition. This modification of subjective peripersonal space was especially pronounced for high caloric food items in the hungry state. We suggest that the observed increase of subjective representation of peripersonal space in a hungry state might be due to the desire to interact with the food items.

We did not find a reliable effect of our valence manipulation on perceived peripersonal space. One possible explanation for this lack of valence effect could be the smaller ratio of high vs. neutral valence ratings (1.40) compared to the ratio of high vs. low calorie ratings (5.87). However, we do not think the lack of valence effect is due to such smaller ratios: The selection criteria for our valence manipulation were based on previous work (Schacht et al., 2016) which used a similar ratio (1.3) for neutral and positive valence values with mean = 48.73 (standard deviation = 3.57) vs. mean = 62.28 (standard deviation = 4.88) from the same database (Blechert et al., 2014); they found a significant difference.

Previous studies have consistently shown object-related influences on peripersonal space representation (Carello et al., 1989; Coello et al., 2003, 2008, 2012; Coello & Delevoye-Turrell, 2007; Delevoye-Turrell et al., 2010; Fischer, 2000, 2005). They suggest that currently available objects and their perceived affordances (e.g., for manipulating the object) are crucial for the subjective perception of peripersonal space. Our results highlight the possibility that also the characteristics of food and the observer’s physiological state influence the subjective perception of peripersonal space. Food deprivation is very likely to be associated with a stronger motivation to seek food, due to the biological drive to restore depleted energy levels (e.g., Anselme & Güntürkün, 2019). This motivation was especially evident for items with a high caloric content. That observation is consistent with Goldfield and Epstein (2002) who have shown a particularly strong willingness to invest effort into obtaining food when the calorie content is high (see also Killgore et al., 2003).

Other recent psychophysical studies have looked at the influence of contextual information on food perception generally (e.g., Revol et al., 2019; Vicario et al., 2019; Zitron-Emanuel & Ganel, 2018). However, the present study offers novel insights into how specific food features, such as their caloric content, change people’s assessment of their action capacities by modifying the subjective perception of their peripersonal space. Nevertheless, the scope of behavioral results alone is limited because they cannot reveal the underlying mechanisms. For this reason, we conducted a second experiment including ERP recordings, with the aim to obtain valuable neural information about the motivational and attentional relevance that may vary with the spatial location of the food pictures.

### ERPs measurements

The ERP results indicate larger amplitudes of the P1 component between 116–140 ms items were displayed at far, relative to near space, suggesting increased early attention allocation for items presented at the far space. Consistent with this interpretation, P1 showed higher amplitudes when a visual stimulus was displayed in the area that the participant was attending (Mangun & Hillyard, 1991; Mangun, 1995). Contrary to Schacht and colleagues (2016), however, we did not find a significant difference between categories of food, e.g. high- vs. neutral-valence, in the P1 amplitude.

With respect to the late positive component (LPC), we found significantly larger amplitudes during hungry than satiated states, in line with previous reports (e.g. Nijs et al., 2008; Schacht et al., 2016; Stockburger et al., 2008, 2009). In terms of location (far space vs. near space), items located at the far distance induced larger LPC amplitudes in comparison with the ones at the near distance. These data seem to suggest that, if motivation is high, the far away items draw more attention than the easily available ones, possibly in order to prepare the individual to make an extra effort for obtaining these food items.

Our findings do not support the report of Valdés-Conroy et al. (2014) that near objects elicited larger ERP amplitudes than far objects. A possible explanation for this discrepancy has to do with the idea that attention may be focused on the distal location instead of proximal space when participants are hungry. Specifically, we suggest that hunger induces a physiological state of necessity, which increases the motivation to obtain food to restore the depleted levels of energy. In order to make far away food items more available participants focus their attention into that area but the larger distance also increases their uncertainty about the outcome of the intended feeding actions. This hypothesis is consistent with previous research which showed that individuals are guided by the expected value of the different outcomes, by the associated degrees of uncertainty (Tobler et al., 2007), and by the prediction of reward when initiating an approach behavior towards that reward (Schultz, 2016). Moreover, with Gigliotti et al. (2019), who revealed that an expected reward in the environment modifies peripersonal space representation. Furthermore, the hypothesis is in agreement with the idea of Kringelbach and Berridge (2017) who highlighted that incentive salience increases the availability of a reward, which helps to determine its motivational value and serves as a potent trigger of ‘wanting’. It is also in line with Blechert et al. (2016) who showed that when food items are available, they are more rewarding. Finally, it is consistent with Guitart-Masip et al. (2014), who suggested that the selection of actions is influenced by the anticipation of the attractiveness of predicted outcomes that promise a reward, and with Coello et al. (2018), who found that subjective perception of peripersonal space increases when the reward-yielding targets are located at far distances.

### Subjective food preference ratings

Both experiments showed that food items were considered as more pleasant when participants were hungry rather than satiated, at the same time high-valence food pictures were rated as more positive than neutral ones. These findings are consistent with those of Rogers & Hardman (2015), who suggest that being hungry and preferring a particular food contributes to the desire to eat. They also match with previous observations (Epstein et al., 2003; Finlayson & Dalton, 2012), showing lower pleasantness ratings after a meal for most food categories. The pleasure translated to hedonic assessment is highly subjective and driven by the knowledge and experience of each individual (Brunyé et al., 2013). According to Epstein et al. (2003) and Kringelbach (2004), subjective feelings related to food refer to pleasure, which derives from eating experiences. According to Finlayson et al. (2008), reported food preferences are problematic because they predict choices for certain foods that participants will not necessarily make. Consistent with that, in our study the subjective rating did not fully converge with the objective reachability errors in which no effect modulated by valence was found.

## Conclusions

Taken together, this paper suggests that incentive-related salience increases the subjective peripersonal space of particular food items when participants are hungry. We also show an attentional enhancement, reflected in early and late ERP components, in far space. We hypothesize that attention seems to be oriented more strongly to far space items due to their expected incentive-related salience, and that this might reflect an intention to reach for these food items.

However, it remains unclear whether the observed changes in peripersonal space are perceptual or motivational in nature. For example, a change of instruction from “imagine to reach” to “would you want to grab” could induce similar changes in perceived peripersonal space, and the ERP findings would not be able to distinguish a motivational from a perceptual interpretation. Thus, our preliminary interpretation of the results in terms of perceptual modification of peripersonal space must be cautious, and our conclusions must await further corroboration.

Moreover, our study had some limitations, such as the absence of non-food stimuli. Our prediction would be that non-food items will not be affected by hungry/satiated manipulations, showing similar subjective peripersonal perception in both conditions, so they could be used as a baseline. It is suggested that more studies need to be conducted in order to accurately substantiate the nature of these results and to be able to apply it in the future to people with obesity and eating disorders.

## Data Accessibility Statement

The data is available on the Open Science Framework at *https://osf.io/y2qz6*.

## Additional File

The additional file for this article can be found as follows:

10.5334/joc.148.s1 Supplementary material. Detailed information of the linear mixed model analysis.

## Footnotes

Because participant and items variables were correlated, a crossed effect mixed model (fixed and random effects) was performed (using the open-source program R and RStudio), using the package lme4 (Bates et al., 2015) with the aim to find a maximal random effect’s structure that could be applied to the data to produce generalizable results (Barr et al., 2013). An additional file with more detailed information of the analysis is available in the supplementary material.

## References

[1] Anselme, P., & Güntürkün, O. (2019). How foraging works: Uncertainty magnifies food-seeking motivation. Behavioral and Brain Sciences, 42, E35. DOI: 10.1017/S0140525X18000948 29514723

[2] Barr, D. J., Levy, R., Scheepers, C., & Tily, H. J. (2013). Random effects structure for confirmatory hypothesis testing: Keep it maximal. Journal of memory and language, 68(3). DOI: 10.1016/j.jml.2012.11.001 PMC3881361 24403724

[3] Bates, D., Mächler, M., Bolker, B., & Walker, S. (2015). “Fitting Linear Mixed-Effects Models Using lme4.” Journal of Statistical Software, 67(1), 1–48. DOI: 10.18637/jss.v067.i01

[4] Berg, P., & Scherg, M. (1994). A multiple source approach to the correction of eye artifacts. Electroencephalography and Clinical Neurophysiology, 90(3), 229–241. DOI: 10.1016/0013-4694(94)90094-9 7511504

[5] Berti, A., & Frassinetti, F. (2000). When far becomes near: Remapping of space by tool use. Journal of Cognitive Neuroscience, 12(3), 415–420. DOI: 10.1162/089892900562237 10931768

[6] Blechert, J., Klackl, J., Miedl, S. F., & Wilhelm, F. H. (2016). To eat or not to eat: Effects of food availability on reward system activity during food picture viewing. Appetite, 99, 254–261. DOI: 10.1016/j.appet.2016.01.006 26796027

[7] Blechert, J., Meule, A., Busch, N. A., & Ohla, K. (2014). Food-pics: An image database for experimental research on eating and appetite. Frontiers in Psychology, 5. DOI: 10.3389/fpsyg.2014.00617 PMC4067906 25009514

[8] Blundell, J., Graaf, C. D., Hulshof, T., Jebb, S., Livingstone, B., Lluch, A., Mela, D., Salah, S., Schuring, E., Knaap, H. V. D., & Westerterp, M. (2010). Appetite control: Methodological aspects of the evaluation of foods. Obesity Reviews, 11(3), 251–270. DOI: 10.1111/j.1467-789X.2010.00714.x 20122136 PMC3609405

[9] Bourgeois, J., Farnè, A., & Coello, Y. (2014). Costs and benefits of tool-use on the perception of reachable space. Acta Psychologica, 148, 91–95. DOI: 10.1016/j.actpsy.2014.01.008 24486811

[10] Brain Vision Analyzer Support Tip—ICA demystified. (2014, 7 10). Brain Products Press Release. https://pressrelease.brainproducts.com/independent-component-analysis-demystified/

[11] Brunyé, T. T., Hayes, J. F., Mahoney, C. R., Gardony, A. L., Taylor, H. A., & Kanarek, R. B. (2013). Get in my belly: Food preferences trigger approach and avoidant postural asymmetries. PloS one, 8(8), e72432. DOI: 10.1371/journal.pone.0072432 24023618 PMC3758305

[12] Carello, C., Grosofsky, A., Reichel, F. D., Solomon, H. Y., & Turvey, M. T. (1989). Visually perceiving what is reachable. Ecological Psychology, 1(1), 27–54. DOI: 10.1207/s15326969eco0101_3

[13] Coello, Y., Richaud, S., Magne, P., & Rossetti, Y. (2003). Vision for spatial perception and vision for action: A dissociation between the left–right and near–far dimensions. Neuropsychologia, 41(5), 622–633. DOI: 10.1016/S0028-3932(02)00200-2 12559155

[14] Coello, Y., Bartolo, A., Amiri, B., Devanne, H., Houdayer, E., & Derambure, P. (2008). Perceiving what is reachable depends on motor representations: Evidence from a transcranial magnetic stimulation study. PLOS ONE, 3(8), e2862. DOI: 10.1371/journal.pone.0002862 18682848 PMC2483935

[15] Coello, Y., Bourgeois, J., & Iachini, T. (2012). Embodied perception of reachable space: How do we manage threatening objects? Cognitive Processing, 13(1), 131–135. DOI: 10.1007/s10339-012-0470-z 22806660

[16] Coello, Y., & Delevoye-Turrell, Y. (2007). Embodiment, spatial categorisation and action. Consciousness and Cognition, 16(3), 667–683. DOI: 10.1016/j.concog.2007.07.003 17728152

[17] Coello, Y., Quesque, F., Gigliotti, M.-F., Ott, L., & Bruyelle, J.-L. (2018). Idiosyncratic representation of peripersonal space depends on the success of one’s own motor actions, but also the successful actions of others! PLoS ONE, 13(5). DOI: 10.1371/journal.pone.0196874 PMC5957367 29771982

[18] Delevoye-Turrell, Y., Bartolo, A., & Coello, Y. (2010). Motor representations and the perception of space: Perceptual judgments of the boundary of action space. Oxford University Press. https://www.oxfordscholarship.com/view/10.1093/acprof:oso/9780199551118.001.0001/acprof-9780199551118-chapter-12. DOI: 10.1093/acprof:oso/9780199551118.003.0012

[19] Derryberry, D., & Tucker, D. M. (1994). Motivating the focus of attention. In the heart’s eye: Emotional influences in perception and attention (pp. 167–196). Academic Press. DOI: 10.1016/B978-0-12-410560-7.50014-4

[20] Dummel, S., & Hübner, R. (2017). Too Tasty to Be Ignored. Experimental Psychology, 64(5), 338–345. DOI: 10.1027/1618-3169/a000373 29173141

[21] Epstein, L. H., Truesdale, R., Wojcik, A., Paluch, R. A., & Raynor, H. A. (2003). Effects of deprivation on hedonics and reinforcing value of food. Physiology & Behavior, 78(2), 221–227. DOI: 10.1016/S0031-9384(02)00978-2 12576119

[22] Finlayson, G., & Dalton, M. (2012). Current progress in the assessment of ‘liking’ vs. ‘wanting’ food in human appetite. Comment on ‘“You say it’s liking, I say it’s wanting…”. On the difficulty of disentangling food reward in man.’ Appetite, 58(1), 373–378. DOI: 10.1016/j.appet.2011.10.011 22057002

[23] Finlayson, G., King, N., & Blundell, J. (2008). The role of implicit wanting in relation to explicit liking and wanting for food: Implications for appetite control. Appetite, 50(1), 120–127. DOI: 10.1016/j.appet.2007.06.007 17655972

[24] Fischer, M. H. (2000). Estimating reachability: Whole body engagement or postural stability? Human Movement Science, 19(3), 297–318. DOI: 10.1016/S0167-9457(00)00016-6

[25] Fischer, M. H. (2005). Action simulation for others is not constrained by one’s own postures. Neuropsychologia, 43(1), 28–34. DOI: 10.1016/j.neuropsychologia.2004.06.003 15488902

[26] Gigliotti, M. F., Soares Coelho, P., Coutinho, J., & Coello, Y. (2019). Peripersonal space in social context is modulated by action reward, but differently in males and females. Psychological Research. DOI: 10.1007/s00426-019-01242-x 31493049

[27] Goldfield, G. S., & Epstein, L. H. (2002). Can fruits and vegetables and activities substitute for snack foods? Health Psychology, 21(3), 299–303. DOI: 10.1037/0278-6133.21.3.299 12027037

[28] Guitart-Masip, M., Duzel, E., Dolan, R., & Dayan, P. (2014). Action versus valence in decision making. Trends in Cognitive Sciences, 18(4), 194–202. DOI: 10.1016/j.tics.2014.01.003 24581556 PMC3989998

[29] Handy, T. C., Grafton, S. T., Shroff, N. M., Ketay, S., & Gazzaniga, M. S. (2003). Graspable objects grab attention when the potential for action is recognized. Nature Neuroscience, 6(4), 421–427. DOI: 10.1038/nn1031 12640459

[30] IBM Knowledge Center. (2014, 10 24). www.ibm.com/support/knowledgecenter/en/sslvmb_24.0.0/spss/regression/idh_prob.html

[31] Iriki, A., Tanaka, M., Obayashi, S., & Iwamura, Y. (2001). Self-images in the video monitor coded by monkey intraparietal neurons. Neuroscience Research, 40(2), 163–173. DOI: 10.1016/S0168-0102(01)00225-5 11377755

[32] Killgore, W. D. S., Young, A. D., Femia, L. A., Bogorodzki, P., Rogowska, J., & Yurgelun-Todd, D. A. (2003). Cortical and limbic activation during viewing of high- versus low-calorie foods. NeuroImage, 19(4), 1381–1394. DOI: 10.1016/S1053-8119(03)00191-5 12948696

[33] Kringelbach M. L. (2004). Food for thought: hedonic experience beyond homeostasis in the human brain. Neuroscience, 126(4), 807–819. DOI: 10.1016/j.neuroscience.2004.04.035 15207316

[34] Kringelbach, M. L., & Berridge, K. C. (2017). Neuroscience of Reward, Motivation, and Drive. Recent Developments in Neuroscience Research on Human Motivation. 19, 23–35. DOI: 10.1108/S0749-742320160000019020

[35] Lang, P. J., & Bradley, M. M. (2010). Emotion and the motivational brain. Biological Psychology, 84(3), 437–450. DOI: 10.1016/j.biopsycho.2009.10.007 19879918 PMC3612949

[36] Linkenauger, S. A., Witt, J. K., Stefanucci, J. K., Bakdash, J. Z., & Proffitt, D. R. (2009). The effects of handedness and reachability on perceived distance. Journal of Experimental Psychology: Human Perception and Performance, 35(6), 1649–1660. DOI: 10.1037/a0016875 19968426 PMC3291021

[37] Mangun, G. R., & Hillyard, S. A. (1991). Modulations of sensory-evoked brain potentials indicate changes in perceptual processing during visual-spatial priming. Journal of Experimental Psychology. Human Perception and Performance, 17(4), 1057–1074. DOI: 10.1037//0096-1523.17.4.1057 1837297

[38] Mangun, G. R. (1995). Neural mechanisms of visual selective attention. Psychophysiology, 32(1), 4–18. DOI: 10.1111/j.1469-8986.1995.tb03400.x 7878167

[39] Meule, A., Kübler, A., & Blechert, J. (2013). Time course of electrocortical food-cue responses during cognitive regulation of craving. Frontiers in Psychology, 4. DOI: 10.3389/fpsyg.2013.00669 PMC3786233 24098290

[40] Meule, A., Lender, A., Richard, A., Dinic, R., & Blechert, J. (2019). Approach–avoidance tendencies towards food: Measurement on a touchscreen and the role of attention and food craving. Appetite, 137, 145–151. DOI: 10.1016/j.appet.2019.03.002 30851311

[41] Neurobehavioral Systems. (n.d.). Retrieved 11 1, 2019, from https://www.neurobs.com/menu_presentation/menu_features/features_overview

[42] Nijs, I. M. T., Franken, I. H. A., & Muris, P. (2008). Food cue-elicited brain potentials in obese and healthy-weight individuals. Eating Behaviors, 9(4), 462–470. DOI: 10.1016/j.eatbeh.2008.07.009 18928910

[43] Nijs, I. M. T., Muris, P., Euser, A. S., & Franken, I. H. A. (2010). Differences in attention to food and food intake between overweight/obese and normal-weight females under conditions of hunger and satiety. Appetite, 54(2), 243–254. DOI: 10.1016/j.appet.2009.11.004 19922752

[44] Ohla, K., Toepel, U., le Coutre, J., & Hudry, J. (2012). Visual-gustatory interaction: Orbitofrontal and insular cortices mediate the effect of high-calorie visual food cues on taste Pleasantness. PLOS ONE, 7(3), e32434. DOI: 10.1371/journal.pone.0032434 22431974 PMC3303800

[45] Oldfield, R. C. (1971). The assessment and analysis of handedness: The Edinburgh inventory. Neuropsychologia, 9(1), 97–113. DOI: 10.1016/0028-3932(71)90067-4 5146491

[46] Papies, E. K., Stroebe, W., & Aarts, H. (2008). The allure of forbidden food: On the role of attention in self-regulation. Journal of Experimental Social Psychology, 44(5), 1283–1292. DOI: 10.1016/j.jesp.2008.04.008

[47] Pegna, A. J., Petit, L., Caldara-Schnetzer, A.-S., Khateb, A., Annoni, J.-M., Sztajzel, R., & Landis, T. (2001). So near yet so far: Neglect in far or near space depends on tool use. Annals of Neurology, 50(6), 820–822. DOI: 10.1002/ana.10058 11761484

[48] Pinheiro, J. C., & Bates, D. M. (2000). Mixed-effects models in S and S-plus. New York: Springer. DOI: 10.1007/978-1-4419-0318-1

[49] Piech, R. M., Lewis, J., Parkinson, C. H., Owen, A. M., Roberts, A. C., Downing, P. E., & Parkinson, J. A. (2009). Neural correlates of appetite and hunger-related evaluative judgments. PLoS ONE, 4(8). DOI: 10.1371/journal.pone.0006581 PMC2719810 19672296

[50] Postman, L., & Bruner, J. S. (1946). The reliability of constant errors in psychophysical measurement. The Journal of Psychology, 21(2), 293–299. DOI: 10.1080/00223980.1946.9917288 21027261

[51] Revol, P., Collette, S., Boulot, Z., Foncelle, A., Niki, C., Thura, D., Imai, A., Jacquin-Courtois, S., Cabanac, M., Osiurak, F., & Rossetti, Y. (2019). Thirst for intention? Grasping a glass Is a thirst-controlled action. Frontiers in Psychology, 10. DOI: 10.3389/fpsyg.2019.01248 PMC6558183 31214073

[52] Rizzolatti, G., Fadiga, L., Fogassi, L., & Gallese, V. (1997). The space around us. Science, 277(5323), 190–191. DOI: 10.1126/science.277.5323.190 9235632

[53] Rizzolatti, G., Scandolara, C., Matelli, M., & Gentilucci, M. (1981). Afferent properties of periarcuate neurons in macaque monkeys. II. Visual responses. Behavioural Brain Research, 2(2), 147–163. DOI: 10.1016/0166-4328(81)90053-X 7248055

[54] Rogers, P. J., & Hardman, C. A. (2015). Food reward. What it is and how to measure it. Appetite, 90, 1–15. DOI: 10.1016/j.appet.2015.02.032 25728883

[55] Schacht, A., Łuczak, A., Pinkpank, T., Vilgis, T., & Sommer, W. (2016). The valence of food in pictures and on the plate: Impacts on brain and body. International Journal of Gastronomy and Food Science, 5–6, 33–40. DOI: 10.1016/j.ijgfs.2016.11.002

[56] Schultz, W. (2016). Dopamine reward prediction error coding. Dialogues in Clinical Neuroscience, 18(1), 23. DOI: 10.31887/DCNS.2016.18.1/wschultz 27069377 PMC4826767

[57] Schupp, H. T., Flaisch, T., Stockburger, J., & Junghöfer, M. (2006). Emotion and attention: Event-related brain potential studies. In S. Anders, G. Ende, M. Junghofer, J. Kissler, & D. Wildgruber (Eds.), Progress in Brain Research, 156, 31–51. Elsevier. DOI: 10.1016/S0079-6123(06)56002-9 17015073

[58] Schur, E. A., Kleinhans, N. M., Goldberg, J., Buchwald, D., Schwartz, M. W., & Maravilla, K. (2009). Activation in brain energy regulation and reward centers by food cues varies with choice of visual stimulus. Int J Obes (Lond). 2009 6; 33(6): 653–61. DOI: 10.1038/ijo.2009.56 19365394 PMC2697279

[59] Seibt, B., Häfner, M., & Deutsch, R. (2007). Prepared to eat: How immediate affective and motivational responses to food cues are influenced by food deprivation. European Journal of Social Psychology, 37(2), 359–379. DOI: 10.1002/ejsp.365

[60] Shapiro, L. (2019). Conceptions of embodiment. Routledge: Embodied Cognition. DOI: 10.4324/9781315180380-4

[61] Stein, S., Lamos, E., Quartuccio, M., Chandraskaran, S., Ionica, N., & Steinle, N. (2013). Food intake and food preference. In V. R. Preedy, L.-A. Hunter, & V. B. Patel (Eds.), Diet Quality: An Evidence-Based Approach, 1, 13–25. Springer. DOI: 10.1007/978-1-4614-7339-8_2

[62] Stockburger, J., Schmälzle, R., Flaisch, T., Bublatzky, F., & Schupp, H. T. (2009). The impact of hunger on food cue processing: An event-related brain potential study. NeuroImage, 47(4), 1819–1829. DOI: 10.1016/j.neuroimage.2009.04.071 19409497

[63] Stockburger, J., Weike, A. I., Hamm, A. O., & Schupp, H. T. (2008). Deprivation selectively modulates brain potentials to food pictures. Behavioral Neuroscience, 122(4), 936–942. DOI: 10.1037/a0012517 18729647

[64] Tanner, D., Morgan-Short, K., & Luck, S. J. (2015). How inappropriate high-pass filters can produce artifactual effects and incorrect conclusions in ERP studies of language and cognition. Psychophysiology, 52(8), 997–1009. DOI: 10.1111/psyp.12437 25903295 PMC4506207

[65] Tobler, P. N., O’Doherty, J. P., Dolan, R. J., & Schultz, W. (2007). Reward value coding distinct from risk attitude-related uncertainty coding in human reward systems. Journal of Neurophysiology, 97(2), 1621–1632. DOI: 10.1152/jn.00745.2006 17122317 PMC2637604

[66] Valdés-Conroy, B., Sebastián, M., Hinojosa, J. A., Román, F. J., & Santaniello, G. (2014). A close look into the near/far space division: A real-distance ERP study. Neuropsychologia, 59, 27–34. DOI: 10.1016/j.neuropsychologia.2014.04.009 24747210

[67] Vicario, C. M., Kuran, K. A., & Urgesi, C. (2019). Does hunger sharpen senses? A psychophysics investigation on the effects of appetite in the timing of reinforcement-oriented actions. Psychological Research, 83(3), 395–405. DOI: 10.1007/s00426-017-0934-y 29086022

[68] Wang, L., Gillis-Smith, S., Peng, Y., Zhang, J., Chen, X., Salzman, C. D., Ryba, N. J. P., & Zuker, C. S. (2018). The coding of valence and identity in the mammalian taste system. Nature, 558(7708), 127–131. DOI: 10.1038/s41586-018-0165-4 29849148 PMC6201270

[69] Weymar, M., Schwabe, L., Löw, A., & Hamm, A. O. (2012). Stress sensitizes the brain: Increased processing of unpleasant pictures after exposure to acute stress. Journal of Cognitive Neuroscience, 24(7), 1511–1518. DOI: 10.1162/jocn_a_00174 22185496

[70] Zitron-Emanuel, N., & Ganel, T. (2018). Food deprivation reduces the susceptibility to size-contrast illusions. Appetite, 128, 138–144. DOI: 10.1016/j.appet.2018.06.006 29885383

