# Supplementary material for: Reaching Out for Food: How Food Incentives Modulate Peripersonal Space Perception
Source: J Cogn. 2021 Mar 10;4(1):21. doi: 10.5334/joc.148 (PMC7954190; doi:10.5334/joc.148)
Supplement: Supplementary material. — Detailed information of the linear mixed model analysis. [file joc-4-1-148-s1.pdf]

Supplementary material for

Reaching out for Food: How Food Incentives Modulate Peripersonal Space Perception.

Matias Bertonatti[1], Mathias Weymar[2,3], Werner Sommer[1], & Martin H. Fischer[4]

1 – Institut für Psychologie, Humboldt-Universität zu Berlin.

2 - Department of Biological Psychology and Affective Science, Faculty of Human Sciences, University of Potsdam, Germany

3 – Faculty of Health Sciences Brandenburg, University of Potsdam, Germany

4 - Department of Cognitive Sciences, University of Potsdam, Germany

Supporting supplementary material:

Mixed model analysis

Because participant and item variables were correlated, a crossed effect mixed model (fixed and random effects) was analyzed (Baayen, Davidson, & Bates, 2008), using the open-source programs R and RStudio with the package lme4 (Bates et al., 2015). Moreover, maximal random effects structure was performed because it has the best potential to produce generalizable results (Barr et al., 2013).

According to Barr et al. (2013, p.11): "*The general principle is that a by-subject (or by-item) random intercept is needed whenever there is more than one observation per subject (or item or subject-item combination), and a random slope is needed for any effect where there is more than one observation for each unique combination of subject and treatment level (or item and treatment level, or subject-item combination and treatment level)". Note:* In our study we used condition (hungry vs satiated) instead of treatment.

- Baayen, R.H., Davidson, D.J., Bates, D.M. (2008). Mixed-effects modeling with crossed random effects for Partiipants and items. Journal of Memory and Language, 59 (2008), pp. 390-412
- Barr, D. J., Levy, R., Scheepers, C., & Tily, H. J. (2013). Random effects structure for confirmatory hypothesis testing: Keep it maximal. Journal of memory and language, 68(3), 10.1016/j.jml.2012.11.001. https://doi.org/10.1016/j.jml.2012.11.001
- Bates D, Mächler M, Bolker B, Walker S (2015). "Fitting Linear Mixed-Effects Models Using lme4." Journal of Statistical Software, 67(1), 1–48. doi: 10.18637/jss.v067.i01.

Reachability Experiment

Because the reachability task involves binary responses (reached/no reached), a mixed effect logistic regression (MELR) was performed. To fit a MELR model in the lme4 package, we used the glmer() function (generalized linear mixed effects regression), with a family=binomial() argument.

Response logit(P(y_{si}=1)) was the dependant variable (binary) (1/0 = reached/no reached)

Crossed random effects:

- Participants (ID) (1,...,s)
- Items (1,...,i)

We used these fixed effect terms (X)

- Condition (Condition_code) (Hungry – Satiated) and its interaction with Positions (Position_cent) (1 to 7)

<u>In lme4 the formula was</u>:

*glmer (Response ~ Condition_code*Position_cent + (1 + Condition_code *Position_cent|ID) + (1 + Condition_code*Position_cent|Items), family="binomial", control=glmerControl(optimizer = "bobyqa")*

The family='binomial' argument specifies this regression uses a "logit link", as for logistic regressions (see R. Baayen et al., 2008). The control=glmerControl(optimizer = "bobyqa") option often facilitates model convergence.

Reachability - Experiment 1

Note: We tried first to fit the model with the crossed random effects of participants and Items and estimate the random slopes of condition interacting with position, per subject and per item with this structure: (1+Condition_code*Position_cent|ID) + (1+Condition_code*Position_cent|Items). However, this model failed to converge. Barr et al. (2013) suggest to stepwise down the complexity of the random effects until the model converges. Thus, to fit the model, we removed the interaction and removed one random slope: (1+Condition_code|ID)+(1|Items). Then, the model converged.

```
Generalized linear mixed model fit by maximum likelihood (Laplace
Approximation) ['glmerMod']
 Family: binomial  ( logit )
Formula: Response ~ Condition_code * Position_cent + (1 +
Condition_code |       ID) + (1 | Items)
   Data: REACH_EXP1
Control: glmerControl(optimizer = "bobyqa")

     AIC      BIC   logLik deviance df.resid
  6642.8   6702.3  -3313.4   6626.8    12592

Scaled residuals:
    Min      1Q  Median      3Q     Max
-81.105  -0.215  -0.005   0.231  74.613

Random effects:
 Groups Name           Variance Std.Dev. Corr
 Items  (Intercept)    0.0403   0.2007
 ID     (Intercept)    3.8455   1.9610
        Condition_code 1.3619   1.1670   0.07
Number of obs: 12600, groups:  Items, 60; ID, 15

Fixed effects:
                             Estimate Std. Error z value Pr(>|z|)
(Intercept)                  -0.05495    0.50799  -0.108   0.9139
Condition_code                1.77281    0.30946   5.729 1.01e-08 ***
Position_cent                -1.75851    0.03337 -52.692  < 2e-16 ***
Condition_code:Position_cent  0.15946    0.06607   2.414   0.0158 *
---
Signif. codes:  0 '***' 0.001 '**' 0.01 '*' 0.05 '.' 0.1 ' ' 1

Correlation of Fixed Effects:
            (Intr) Cndtn_ Pstn_c
Conditin_cd  0.066
Positin_cnt  0.004 -0.101
Cndtn_cd:P_ -0.031  0.012 -0.104
```

Reachability - Experiment 2

Note: We tried first to fit the model with the crossed random effects of participants and Items and estimate the random slopes of condition interacting with position, per subject and per item with this structure: (1+Condition_code*Position_cent | ID) + (1+Condition_code*Position_cent | Items). However, this model failed to converge. Barr et al. (2013) suggest to stepwise the complexity of the random effects until the model converges. As for Experiment 1, we again followed the step wise procedure suggested by Barr et al. (2013). To fit the model, we removed the interaction (Condition*Position), and we tried with (1+Condition_code|ID)+(1+ Condition_code | Items). Then, the model converged.

```
Generalized linear mixed model fit by maximum likelihood (Laplace
Approximation) ['glmerMod']
 Family: binomial  ( logit )
Formula: Response ~ Condition_code * Position_cent + (1 +
Condition_code |      ID) + (1 + Condition_code | Items)
   Data: REACH_EXP2
Control: glmerControl(optimizer = "bobyqa")

     AIC      BIC   logLik deviance df.resid
 10020.8  10098.9  -5000.4  10000.8    18190

Scaled residuals:
     Min       1Q   Median       3Q      Max
-108.119   -0.235   -0.024    0.234   39.686

Random effects:
 Groups Name           Variance Std.Dev. Corr
 Items  (Intercept)    0.15276  0.3908
        Condition_code 0.08798  0.2966   -0.37
 ID     (Intercept)    2.72339  1.6503
        Condition_code 3.66634  1.9148   0.21
Number of obs: 18200, groups:  Items, 60; ID, 22

Fixed effects:
                             Estimate Std. Error z value Pr(>|z|)
(Intercept)                  -0.19736    0.35654  -0.554     0.58
Condition_code                1.94904    0.41399   4.708 2.50e-06 ***
Position_cent                -1.69207    0.02664 -63.526  < 2e-16 ***
Condition_code:Position_cent -0.24853    0.05327  -4.665 3.08e-06 ***
---
Signif. codes:  0 '***' 0.001 '**' 0.01 '*' 0.05 '.' 0.1 ' ' 1

Correlation of Fixed Effects:
            (Intr) Cndtn_ Pstn_c
Conditin_cd  0.197
Positin_cnt  0.003 -0.067
Cndtn_cd:P_ -0.039  0.006  0.170
```

Table 1

Estimated parameters from both Experiment 1 and 2 of logistic mixed-effects regression models (binomial responses). Sjplot package - tab _model(), Lüdecke D (2020)

	**Experiment 1**			**Experiment 2**		
	**Response**			**Response**		
Predictors	*Log-Odds*	*std. Error*	*p*	*Log-Odds*	*std. Error*	*p*
(Intercept)	-0.05	0.51	0.914	-0.20	0.36	0.580
Condition_code	1.77	0.31	**<0.001**	1.95	0.41	**<0.001**
Position_cent	-1.76	0.03	**<0.001**	-1.69	0.03	**<0.001**
Condition_code:Position_cent	0.16	0.07	**0.016**	-0.25	0.05	**<0.001**
**Random Effects**						
σ^2	3.29			3.29		
τ_{00}	0.04 $_{Items}$			0.15 $_{Items}$		
	3.85 $_{ID}$			2.72 $_{ID}$		
τ_{11}	1.36 $_{ID.Condition\_code}$			0.09 $_{Items.Condition\_code}$		
				3.67 $_{ID.Condition\_code}$		
ρ_{01}	0.07 $_{ID}$			-0.37 $_{Items}$		
				0.21 $_{ID}$		
N	15 $_{ID}$			22 $_{ID}$		
	60 $_{Items}$			60 $_{Items}$		
Observations	12600			18200		

τ_{00}=Intercepts variance, τ_{11}=Slopes variance, ρ01=Correlation between pairs (Intercepts – Slopes), σ^{2}=Residual error

Note: For generalized linear models, the output is slightly adapted. Instead of Estimates, the column is named Log-Odds.

Lüdecke D (2020). _sjPlot: Data Visualization for Statistics in Social Science_. R package version 2.8.6, <URL: https://CRAN.R-project.org/package=sjPlot/vignettes/tab_mixed.html >.

Linear mixed model (LMM)

For the Subjective food preference ratings part Linear Mixed Model (LMM) was performed. To fit an LMM in the lme4 package, we used the lmer() function (linear mixed effects regression).

Rating y_{si} is the dependant variable

Crossed random effects:

- Participants (ID) (1,...,s)
- Items (1,...,i)

We used these fixed effect terms (X)

- Condition (Condition_code) (Hungry – Satiated)
- Calorie (Calorie_code) (High – Low)
- Valence (Valence_code) (High – Neutral)

<u>In lme4 the formula was</u>:

lmer (Rating ~ Condition_code + Calorie_code + Valence_code + (1 + Condition_code| ID) + (1 + Condition_code| Items)

Subjective food preference ratings - Experiment 1

```
Linear mixed model fit by REML. t-tests use Satterthwaite's method
['lmerModLmerTest']
Formula: Rating ~ Condition_code + Calorie_code + Valence_code +
(1 +      Condition_code | ID) + (1 + Condition_code | Items)
   Data: SUBJ_RATINGS_EXP1

REML criterion at convergence: 5722.1

Scaled residuals:
    Min      1Q  Median      3Q     Max
-3.4873 -0.7018 -0.0379  0.6779  2.8029

Random effects:
 Groups   Name           Variance Std.Dev. Corr
 Items    (Intercept)    0.07533  0.2745
          Condition_code 0.85957  0.9271   0.26
 ID       (Intercept)    0.19096  0.4370
          Condition_code 0.98394  0.9919   0.08
 Residual                1.21233  1.1011
Number of obs: 1800, groups:  Items, 60; ID, 15

Fixed effects:
               Estimate Std. Error       df t value Pr(>|t|)
(Intercept)     2.78833    0.12108 16.65938  23.029 4.57e-14 ***
Condition_code  1.16111    0.28743 20.19450   4.040 0.000631 ***
Calorie_code    0.14437    0.08623 56.99782   1.674 0.099558 .
Valence_code    0.39785    0.08623 56.99782   4.614 2.29e-05 ***
---
Signif. codes:  0 '***' 0.001 '**' 0.01 '*' 0.05 '.' 0.1 ' ' 1

Correlation of Fixed Effects:
            (Intr) Cndtn_ Clr_cd
Conditin_cd 0.096
Calorie_cod 0.000  0.000
Valence_cod 0.000  0.000  0.000
```

Subjective food preference ratings - Experiment 2

```
Linear mixed model fit by REML. t-tests use Satterthwaite's method
['lmerModLmerTest']
Formula: Rating ~ Condition_code + Calorie_code + Valence_code +
(1 +      Condition_code | ID) + (1 + Condition_code | Items)
   Data: SUBJ_RATINGS_EXP2

REML criterion at convergence: 7247.8

Scaled residuals:
    Min      1Q  Median      3Q     Max
-3.4273 -0.5090 -0.0848  0.6047  3.2804

Random effects:
 Groups   Name           Variance Std.Dev. Corr
 Items    (Intercept)    0.07381  0.2717
          Condition_code 0.27743  0.5267   0.42
 ID       (Intercept)    0.26272  0.5126
          Condition_code 0.31074  0.5574   -0.39
 Residual                0.92458  0.9615
Number of obs: 2520, groups:  Items, 60; ID, 21

Fixed effects:
               Estimate Std. Error       df t value Pr(>|t|)
(Intercept)     2.45833    0.11878 23.86652  20.697  < 2e-16 ***
Condition_code  1.69762    0.14453 31.48504  11.746 4.83e-13 ***
Calorie_code   -0.11321    0.07563 56.99979  -1.497     0.14
Valence_code    0.39003    0.07563 56.99979   5.157 3.28e-06 ***
---
Signif. codes:  0 '***' 0.001 '**' 0.01 '*' 0.05 '.' 0.1 ' ' 1

Correlation of Fixed Effects:
            (Intr) Cndtn_ Clr_cd
Conditin_cd -0.254
Calorie_cod  0.000  0.000
Valence_cod  0.000  0.000  0.000
```

Table 2

Estimated parameters from both Experiment 1 and 2 of Linear Mixed Models.
Sjplot package – tab_model(), Lüdecke D (2020)

	Rating			Rating		
Predictors	*Estimates*	*std. Error*	*p*	*Estimates*	*std. Error*	*p*
(Intercept)	2.79	0.12	**<0.001**	2.46	0.12	**<0.001**
Condition_code	1.16	0.29	**<0.001**	1.70	0.14	**<0.001**
Calorie_code	0.14	0.09	0.094	-0.11	0.08	0.134
Valence_code	0.40	0.09	**<0.001**	0.39	0.08	**<0.001**
**Random Effects**						
σ^2	1.21			0.92		
τ_{00}	0.08 Items			0.07 Items		
	0.19 ID			0.26 ID		
τ_{11}	0.86 Items.Condition_code			0.28 Items.Condition_code		
	0.98 ID.Condition_code			0.31 ID.Condition_code		
ρ_{01}	0.26 Items			0.42 Items		
	0.08 ID			-0.39 ID		
N	15 ID			21 ID		
	60 Items			60 Items		
Observations	1800			2520		

τ_{00}=Intercepts variance, τ_{11}=Slopes variance, ρ_{01}=Correlation between pairs (Intercepts – Slopes), σ^{2}=Residual error

Lüdecke D (2020). _sjPlot: Data Visualization for Statistics in Social Science_. R package version 2.8.6, <URL: https://CRAN.R-project.org/package=sjPlot/vignettes/tab_mixed.html >.

ERP – Analysis
For the ERP part, a Linear Mixed Model (LMM) was performed. To fit an LMM in the lme4 package, we used the lmer() function (linear mixed effects regression).

Amplitude y_{si} is the dependant variable

Crossed random effects:

- Participants (ID) (1,...,s)
- Items (1,...,i)

We used these fixed effect terms (X)

- Condition (Condition_code) (Hungry – Satiated) and its interaction with Distance (Distance_code) (Near - Far)

<u>In lme4 the formula was</u>:

*lmer (Amplitude ~ Condition_code * Distance_code + (1 + Condition_code * Distance_code | ID) + (1 + Condition_code*Distance_code| Items)*

ERP - P1

Note: We tried first to fit the model with the crossed random effects of participants and Items and estimate the random slopes of condition interacting with distance, per subject and per item with this structure: (1+Condition_code*Distance_code|ID) + (1+Condition_code*Distance_code | Items). However, this model failed to converge. Barr et al. (2013) suggest to stepwise down the complexity of the random effects until the model converges. Thus, to fit the model, we removed the interaction and removed one random slope: (1+Condition_code|ID)+(1|Items). Then, the model converged.

```
Linear mixed model fit by REML. t-tests use Satterthwaite's method
['lmerModLmerTest']
Formula: Amplitude_P1 ~ Condition_code * Distance_code + (1 + Condition_code |
ID) + (1 | Items)
   Data: ERP_P1
Control: lmerControl(optimizer = "bobyqa")

REML criterion at convergence: 85885.8

Scaled residuals:
     Min       1Q   Median       3Q      Max
-15.0697  -0.5330  -0.0002   0.5266  10.2048

Random effects:
 Groups   Name           Variance Std.Dev. Corr
 Items    (Intercept)     0.03246 0.1802
 ID       (Intercept)     1.55265 1.2461
          Condition_code  0.39487 0.6284   0.38
 Residual                23.99959 4.8989
Number of obs: 14255, groups:  Items, 60; ID, 22

Fixed effects:
                              Estimate Std. Error        df t value Pr(>|t|)
(Intercept)                  1.873e+00  2.698e-01 2.131e+01   6.942 6.82e-07 ***
Condition_code               3.069e-01  1.573e-01 1.986e+01   1.951   0.0653 .
Distance_code                4.438e-01  8.210e-02 1.416e+04   5.406 6.54e-08 ***
Condition_code:Distance_code 2.890e-01  1.642e-01 1.416e+04   1.760   0.0783 .
---
Signif. codes:  0 '***' 0.001 '**' 0.01 '*' 0.05 '.' 0.1 ' ' 1

Correlation of Fixed Effects:
            (Intr) Cndtn_ Dstnc_
Conditin_cd  0.314
Distance_cd  0.000  0.000
Cndtn_cd:D_  0.000  0.000 -0.023
```

ERP - LPC

Note: We tried first to fit the model with the crossed random effects of participants and Items and estimate the random slopes of condition interacting with distance, per subject and per item with this structure: (1+Condition_code*Distance_code | ID) + (1+Condition_code*Distance_code | Items). However, this model failed to converge. Barr et al. (2013) suggest to stepwise the complexity of the random effects until the model converges. As for the P1 mixed effect analysis, we again followed the step wise procedure suggested by Barr et al. (2013). To fit the model, we removed the interaction (Condition_code*Distance_code), and we tried

```
Linear mixed model fit by REML. t-tests use Satterthwaite's method
['lmerModLmerTest']
Formula: Amplitude_LPC ~ Condition_code * Distance_code + (1 + Condition_code |
ID) + (1 + Condition_code | Items)
   Data: ERP_LPC
Control: lmerControl(optimizer = "bobyqa")

REML criterion at convergence: 85918.2

Scaled residuals:
    Min      1Q  Median      3Q     Max
-7.2239 -0.5557 -0.0032  0.5464 11.0792

Random effects:
 Groups   Name           Variance  Std.Dev. Corr
 Items    (Intercept)     0.092431 0.30402
          Condition_code  0.004047 0.06361  0.78
 ID       (Intercept)     3.317486 1.82140
          Condition_code  1.787475 1.33696  0.50
 Residual                23.950249 4.89390
Number of obs: 14255, groups:  Items, 60; ID, 22

Fixed effects:
                              Estimate Std. Error        df t value Pr(>|t|)
(Intercept)                  3.843e+00  3.925e-01 2.139e+01   9.793 2.33e-09 ***
Condition_code               8.000e-01  2.968e-01 2.070e+01   2.695  0.01366 *
Distance_code                4.893e-01  8.202e-02 1.411e+04   5.966 2.49e-09 ***
Condition_code:Distance_code 4.754e-01  1.640e-01 1.411e+04   2.899  0.00375 **
---
Signif. codes:  0 '***' 0.001 '**' 0.01 '*' 0.05 '.' 0.1 ' ' 1

Correlation of Fixed Effects:
            (Intr) Cndtn_ Dstnc_
Conditin_cd  0.475
Distance_cd  0.000  0.000
Cndtn_cd:D_  0.000  0.000 -0.023
```

with (1+Condition_code|ID)+(1+ Condition_code|Items). Then, the model converged.
